# Bayesian classification of OXPHOS deficient skeletal myofibres

**DOI:** 10.1371/journal.pcbi.1012770

**Published:** 2025-02-19

**Authors:** Jordan Childs, Tiago Bernardino Gomes, Amy E Vincent, Andrew Golightly, Conor Lawless

**Affiliations:** 1 Wellcome Centre for Mitochondrial Research, Newcastle University, Newcastle-upon-Tyne, United Kingdom; 2 Newcastle University Translational and Clinical Research Institute, Newcastle-upon-Tyne, United Kingdom; 3 NIHR Biomedical Research Centre, Newcastle University, Newcastle-upon-Tyne, United Kingdom; 4 NHS Highly Specialised Service for Rare Mitochondrial Disorders, Newcastle-upon-Tyne, United Kingdom; 5 Department of Mathematical Sciences, Durham University, Durham, United Kingdom; University of Waterloo, CANADA

## Abstract

Mitochondria are organelles in most human cells which release the energy required for cells to function. Oxidative phosphorylation (OXPHOS) is a key biochemical process within mitochondria required for energy production and requires a range of proteins and protein complexes. Mitochondria contain multiple copies of their own genome (mtDNA), which codes for some of the proteins and ribonucleic acids required for mitochondrial function and assembly. Pathology arises from genetic defects in mtDNA and can reduce cellular abundance of OXPHOS proteins, affecting mitochondrial function. Due to the continuous turn-over of mtDNA, pathology is random and neighbouring cells can possess different OXPHOS protein abundance. Estimating the proportion of cells where OXPHOS protein abundance is too low to maintain normal function is critical to understanding disease severity and predicting disease progression. Currently, one method to classify single cells as being OXPHOS deficient is prevalent in the literature. The method compares a patient’s OXPHOS protein abundance to that of a small number of healthy control subjects. If the patient’s cell displays an abundance which differs from the abundance of the controls then it is deemed deficient. However, due to the natural variation between subjects and the low number of control subjects typically available, this method is inflexible and often results in a large proportion of patient cells being misclassified. These misclassifications have significant consequences for the clinical interpretation of these data. We propose a single-cell classification method using a Bayesian hierarchical mixture model, which allows for inter-subject OXPHOS protein abundance variation. The model accurately classifies an example dataset of OXPHOS protein abundances in skeletal muscle fibres (myofibres). When comparing the proposed and existing model classifications to manual classifications performed by experts, the proposed model results in estimates of the proportion of deficient myofibres that are consistent with expert manual classifications.

## Author summary

Mitochondria are responsible for energy production within cells and mitochondrial diseases correspond to a group of rare genetic disorders which can affect their function. However, it is common for each cell to not be affected in the same way, and neighbouring cells are often observed with varying levels of mitochondrial function. Estimating the proportion of affected cells in a sample is important for measuring disease progression and severity. One way to measure mitochondrial function is through the abundance of proteins related to their function. Using this data we have developed a method to classify single cells with a mitochondrial defect which allows for the natural variability between subjects. We show that our approach produces classifications in higher agreement with those performed by experts when compared to an existing classification method.

## Introduction

Mitochondria are organelles within eukaryotic cells responsible for producing adenosine triphosphate (ATP), the chemical used to store and release energy. They contain many copies of their own DNA (mtDNA), a state known as polyploidy. MtDNA encodes several proteins as well as mitochondrial transfer and ribosomal ribonucleic acid molecules, essential for mitochondrial function [1]. Depending on cell type, a single cell can contain hundreds of thousands of mtDNA copies [2]. MtDNA polyploidy and continuous mtDNA turnover make mitochondrial genetics quite unusual in that their population dynamics strongly affect the concentration of mitochondrial proteins within the cell [3–5].

Mitochondrial diseases are a group of rare genetic conditions that affect the ability of a mitochondrion to produce ATP through oxidative phosphorylation (OXPHOS) [6]. They are caused by variations in either the nuclear or mitochondrial genomes [7]. Inherited nuclear-encoded variants are present throughout life, at either one or two copies per cell, and are present at the same zygosity in all cells within an organism. Mitochondrial diseases caused by variants in mtDNA are different. The polyploidy of mtDNA allows a wide range of variant proportions to exist within a single cell. For example, in a cell with *N* copies of mtDNA and two mtDNA species: one healthy (wild-type) and one variant, proportions of variant molecules can take any value in the range $0,\frac{1}{N},\frac{2}{N},...,1$. The continuous replication and degradation of mtDNA allows the proportion of variant mtDNA to vary dynamically throughout the life of a single-cell [7–9]. The biochemical threshold theory proposes that mitochondrial function becomes disrupted (acquires an OXPHOS defect) if the variant proportion of mtDNA passes a pathogenic threshold [10]. Variations in mtDNA can impact subunits of the OXPHOS complexes or the translation of these proteins, often leading to a lower abundance of some OXPHOS subunits. Mitochondrial disease symptoms vary depending on the mtDNA variant present and the tissues affected.

Symptoms and variant proportions in children can differ drastically from those in parents (mothers) due to the random segregation of mtDNA populations during the meiotic cell divisions required for the germ line to develop [7]. Symptom severity can also differ between tissues due to mtDNA segregation during mitotic cell division required to develop the soma. MtDNA replication associated with cell division is known as strict replication. Random mtDNA replication occurring throughout cell life (and independent of cell division) is known as relaxed replication [11].

**Fig 1:**
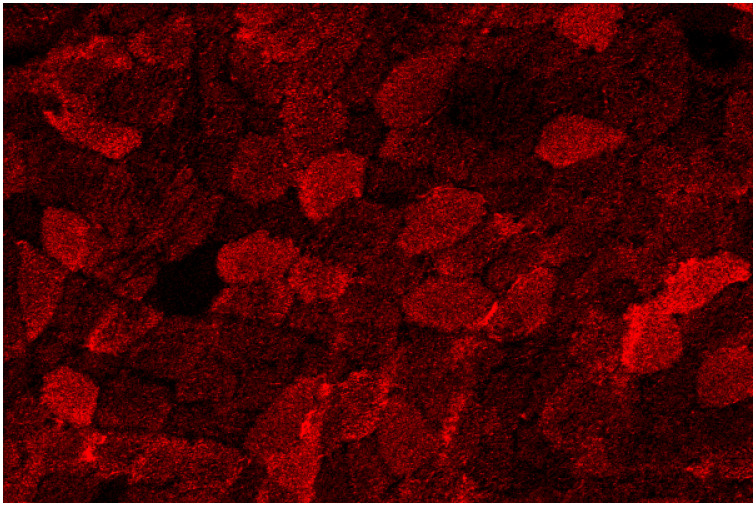
Random, mosaic pattern of single skeletal muscle fibres OXPHOS protein abundance within a cross-section. Pseudo image of approximately 130 skeletal muscle fibre cross-sections captured by imaging mass cytometry after needle biopsy of a patient (P05) with a nuclear DNA variant causing multiple deletions in mtDNA. Yellow shows the level of VDAC (mitochondrial mass surrogate), red shows the level of MTCO1 (OXPHOS protein), and white shows the level of DMD (cell membrane marker).

Segregation and both forms of replication contribute to the stochasticity of mtDNA population dynamics, resulting in adjacent cells possessing very different proportions of variant mtDNA. This results in a random mosaic pattern of healthy and dysfunctional cells within the same tissue [12], as demonstrated in Fig 1.

MtDNA mutation events allow variant mtDNA to be acquired during ageing and can accumulate within a subject without causing mitochondrial disease [13]. The chances of a *de novo* variant population arising that significantly affects tissue function are negligible in healthy subjects. However, it is possible that a variant mtDNA population clonally expands to become the majority population within a single cell [5]. The proportion of cells within a tissue with an OXPHOS defect is an important measure of pathological progression: a higher proportion means fewer cells are functioning normally, and there is an increased likelihood of clinical symptoms and tissue pathology.

Quantifying and characterising the dysfunction of cells within a tissue is not trivial [14]. Single-cell observations of relative protein abundances can be made by cutting cross-sections of tissue samples from healthy control or patient subjects, immunolabelling for a targeted panel of proteins by immunofluorescence (IF) and imaging [14] or by Imaging Mass Cytometry (IMC) [15]. IMC and IF measure the spatial distribution of proteins as pixel intensities within an image of a tissue cross-section. The relative protein abundance in a single cell is calculated by first segmenting single-cell cross-sections from imaging data and calculating the average intensity per segmented cell. The mean intensity is assumed to be proportional to the number of protein molecules within the cross-section, and we refer to this as protein abundance. Previously, both IF and IMC have been validated using sequential cytochrome c oxidate/Succinate Dehydrogenase histochemistry, demonstrating that the protein levels of complex IV proteins are biologically relevant in terms of complex IV activity [14,15].

The dataset we use here is taken from samples of skeletal muscle tissue biopsies from patients with a nuclear-encoded defect in mtDNA replication and maintenance [9,16]. Consequently, the patients are predisposed to rapidly accumulate *de novo* mtDNA variants, compared to natural ageing. We use single skeletal muscle fibre (myofibre) protein abundance data and aim to identify each myofibre within the patient samples which has an OXPHOS defect. A patient myofibre is assumed to have an OXPHOS defect if its OXPHOS protein abundance is unlike that of healthy control subjects [14,15]. Throughout the paper, we use the term *myofibre* as this is the cell type of the example dataset however the methods presented here could be applied to any cell type.

Patients with mitochondrial disease exhibit a mixture of myofibres in their biopsies that are variably affected by disease pathology, as seen in Fig 1. A proportion of these myofibres will be very similar to that of a healthy individual with correctly functioning mitochondria and exhibit a strong positive correlation between the mean intensities of different mitochondrial proteins [14]. In comparison, a subset of myofibres will be impacted by mitochondrial disease pathology, which impacts both the function of the mitochondria and the abundance of OXPHOS proteins. Within this group there will typically be a mix of myofibres, which have a reduction in protein subunits that form OXPHOS complexes I, III, IV and/or V and associated dysfunction of these complexes [14]. Therefore, when we consider the classification of myofibres we can think of splitting these into two groups: like-control, where the mitochondria function and protein abundances are similar to that of a healthy myofibres, and, not-like-control where the myofibres are impacted by one or more changes in OXPHOS subunit abundance and OXPHOS complex activity. Importantly, their relative abundances allow for the identifications of biologically relevant functional and dysfunctional myofibres [14,15].

### Previous work

**Fig 2:**
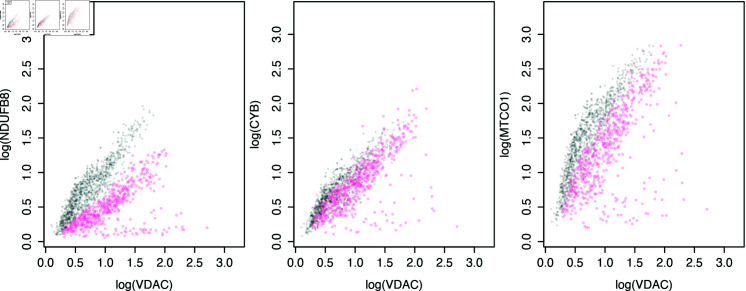
Single-myofibre OXPHOS protein abundances split into two populations; like-control and not-like-control. Single-fibre protein abundances were collected by Imaging Mass Cytometry (IMC) from skeletal muscle myofibres from four healthy control subjects (grey, 1,155 myofibres) and one patient, P09, (pink, 571 myofibres).

One approach to visualise mitochondrial OXPHOS protein abundance profiles is to use a 2Dmito plot. These are 2-dimensional scatter plots of single-myofibre mitochondrial protein abundances, containing many single-myofibre observations from a few healthy control subjects and one patient subject. They illustrate how OXPHOS protein abundance (*y*-axis) varies with mitochondrial mass (*x*-axis). The mitochondrial mass accounts for the variation in mitochondria population size within myofibres. In this dataset, VDAC, a protein found on the outer membrane of mitochondria, is used as a surrogate for mitochondrial mass. Example 2Dmito plots can be seen in Fig 2.

Previously, Rocha *et al.* implemented a classification pipeline based on a frequentist linear regression framework to label each myofibre in a patient tissue sample as like-control or not-like-control [14]. The classification pipeline works as follows. The single-myofibre protein abundances are log-transformed, as suggested by the Box-Cox test. Data from all control subjects are combined into a single dataset. A frequentist linear model is fitted to the single-myofibre measurements of log OXPHOS protein abundance (the response variable) with a single predictor, the corresponding measurements of log mitochondrial mass. The classification is described in Eqs 1 and 2. Here, $\left\{X_{j}^{c},Y_{j}^{c}\right\}$ are the measurements of log mitochondrial mass and log OXPHOS protein abundance of the *j*-th control myofibre. The linear model is


$$\begin{array}{c} Y_{j}^{c}\sim N\left(mX_{j}^{c}+c,\tau^{-1}\right), \end{array}$$ (1)

where $N(\mu ,\tau^{-1})$ denotes a normal distribution with expectation *μ* and precision *τ*. The slope and intercept of the regression model are denoted *m* and *c* respectively.

Rocha *et al.* classify a patient’s myofibre, $\left\{X_{j}^{p},Y_{j}^{p}\right\}$, into four arbitrary groups based upon a continuous Z-score which measures the vertical distance of each myofibre from the model’s expected value [14]. However, Warren *et al.* found that, instead of a continuum of deficiency, most patient OXPHOS protein abundance data could be more naturally split into exactly two myofibre populations: like-control and not-like-control [15]. This two-class split is also consistent with the biochemical threshold theory [10]. Warren *et al.* also found some patients where specific proteins were more abundant than in control subjects and, consequently, implemented a ternary classification [15]. Classifying a patient myofibre as being over- or under-expressed if OXPHOS protein abundance fell above or below the 95% predictive interval of a linear model fitted to the control subjects’ data.

Following the classification model of Warren *et al.*, the *j*^th^ patient myofibre, $\left\{X_{j}^{p},Y_{j}^{p}\right\}$, is classified as being not-like-control if it lies outside the 95% predictive interval at the point $X_{j}^{p}$, computed using the frequentist linear model [15]. Therefore, let the binary variable $Z_{j}$ be


$$\begin{array}{c} Z_{j}=\{\begin{array}{cc} 1\; & Y_{j}^{p}\notin [L_{j},U_{j}],\\ 0\; & otherwise \end{array} \end{array}$$ (2)

indicating that the patient’s *j*-th myofibre possesses an OXPHOS defect if $Z_{j}=1$, $L_{j}$ and $U_{j}$ are the lower and upper bounds of the 95% predictive interval for $X_{j}^{p}$.

The frequentist linear model classification pipeline, proposed by Rocha *et al.* [14], has become prevalent within the literature, with over 120 papers citations since publication, of which more than 25 have used this method directly [17–20]. As we will show in this paper, the rigidity of the frequentist linear model can result in a large proportion of misclassifications. This occurs when the patient’s protein abundance differs in any way from the control’s. We propose a Bayesian hierarchical alternative to the existing frequentist classification pipeline that allows healthy patient myofibres to differ from the controls to tolerate technical and natural variability affecting protein abundance measurements.

Bayesian methods are used throughout the literature in the study of complex biological systems. Their inherent ability to account for uncertainty in model parameters and prediction makes them well-suited for quantifying the natural variation and observational error commonly seen in biological datasets. For methods and applications of Bayesian statistics within biological sciences see [21–26].

### Data and exploratory analysis

#### The data.

The data used here come from skeletal muscle biopsies of 12 patients with genetically and clinically characterised mitochondrial disease caused by variants in their nuclear DNA [27]. Nuclear variants harboured by the patients disrupt mtDNA replication and maintenance, causing mtDNA variants to arise, by mutation events, much more rapidly than by normal ageing. Excess muscle was collected from the hamstring muscle of four healthy control subjects during anterior cruciate ligament surgery. 6 μm tissue cross-sections were cut from the biopsies and assessed using IMC. IMC is a destructive measuring process by which a laser ablates metal-label antibody-stained tissue sections in 1μm^2^ dots with each laser pulse. Metal abundance in the vapour from the ablated area is measured by mass cytometry. The abundance of up to 40 proteins can be measured simultaneously and assembled into a histological pseudo-image (one pixel per pulse) to represent the spatial distribution of protein abundances within and between myofibres. The average protein abundance of each myofibre is calculated as the average signal per protein channel after segmenting the image pixels into individual myofibres using a cell membrane marker (DMD in this case) and the mitocyto segmentation tool [15]. A 2Dmito plot is created from the average protein abundance per myofibre from one patient and all control subjects. The IMC procedure for one patient, P08, failed, and this patient was removed from the dataset.

The dataset includes measurements for a large number of proteins. Here, we restrict analysis to the OXPHOS proteins CYB, NDUFB8, and MTCO1, the latter two being the most frequently used proteins to assess mitochondrial OXPHOS subunits in the literature [9,14,28,29]. VDAC protein data were also included as a surrogate measure of mitochondrial mass. A full description of data collection methods can be found in Vincent *et al.* [27]. The number of myofibres in the resulting healthy control samples ranges from 154 to 363 per section, and the patient samples range between 151 and 1,199.

#### Manual classification.

**Fig 3:**
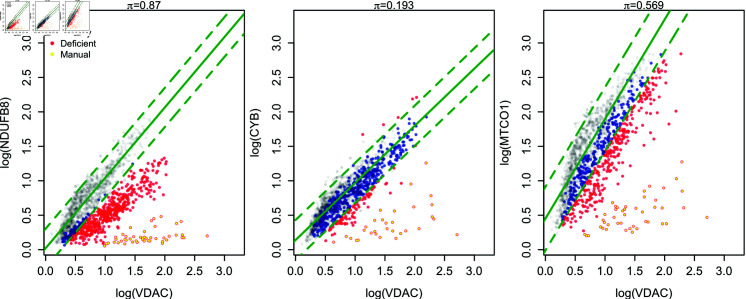
Frequentist classification arbitrarily splits healthy myofibre populations. (top) Frequentist model’s 95% predictive interval and classifications for all three OXPHOS proteins in P09 with 571 myofibres (coloured points). Control myofibres are shown in grey (1,155 from four healthy subjects). Patient myofibres are coloured blue or red, depending if the model classified them as like-control or not-like-control respectively. The 95% predictive interval and fitted values for the model are shown in green. The manually classified not-like-control myofibres are shown with a small yellow dot within the myofibre’s point.

To evaluate model performance three experts manually classified the OXPHOS status of patient myofibres, based on their OXPHOS protein abundances. Each selected the patient myofibres within a 2Dmito plot which they believed to be not-like-control, in the relevant OXPHOS protein. However, experts can disagree on which myofibres are like-control and which are not. Therefore, only myofibres agreed upon by all three were considered not-like-control. An example of the manual classifications can be seen in Fig 3. In this work, we consider the manual classifications as a ground-truth state, against which we compare the existing and proposed classification methods.

#### Data exploration.

Log-transformation of the data was proposed to bring the Rocha *et al.* control subject data in line with assumptions of linear regression, e.g. independent and normally distributed residuals with a constant variance [14]. However, the log transformation does not make a large difference with this particular dataset. Using the Box-Cox power transformation separately on each control subject and OXPHOS protein, we see that no data transform is suggested for control subjects C01, C02 and C03 in proteins NDUFB8 and CYB, and a transformation of approximately $x:\to x^{1.5}$ is suggested for MTCO1. C04 is different, and it is suggested that no transformation is required for MTCO1, but NDUFB8 and CYB are approximately transformed by $x:\to x^{0.75}$. Nevertheless, we log-transformed the protein abundances as this transformation has been shown to drastically reduce non-normality in other similar datasets collected by both IMC and IF and on a range of mitochondrial proteins, including the OXPHOS proteins analysed here [14,15,27].

We aim to classify each patient myofibre as like-control or not-like-control for each OXPHOS protein and quantify the proportion of not-like-control myofibres within each patient sample and OXPHOS protein. Therefore, we must characterise the protein abundance profiles in healthy subjects to identify not-like-control myofibres by comparison. The control data show a strong linear relationship between the mitochondrial mass marker VDAC and each of the OXPHOS proteins. Fig 2 shows example 2Dmito plots for all OXPHOS proteins in patient P09.

We find a population of myofibres lying adjacent to the healthy myofibres from the control subjects, showing a strong linear relationship and a small, separate population below these. It is common to see the second population showing no (or very little) correlation between the OXPHOS protein abundance and mitochondrial mass (VDAC). We define the population of myofibres that show a similar profile to the controls as like-control and the other population as not-like-control. Importantly, the first panel in Fig 2 also demonstrates that the population of healthy myofibres in a patient sample can have slopes and intercepts that are different from the healthy myofibres from control subjects.

#### Existing classification method.

The control subjects show a strong linear relationship between (log) OXPHOS protein abundance and (log) VDAC. Therefore, it is natural to first fit the existing frequentist linear model classification pipeline. Fig 3 shows the classifications of patient P09 in the dataset for all three OXPHOS proteins. Patient myofibres are shown in blue or red, indicating a like-control or not-like-control classification respectively. The manually classified myofibres are highlighted with a yellow dot if they are not-like-control. The quality of the classifications in this patient are very mixed. The model has correctly classified the CYB status of most myofibres. A small number of myofibres in the edge of the group lying adjacent to the controls are incorrectly classified as not-like-control. Classification of the patient’s myofibres MTCO1 status has failed, arbitrarily splitting the manually classified like-control myofibres in two, resulting in many misclassifications. The classification for NDUFB8 has also failed; almost all myofibres have been classified as not-like-control, in contradiction to the manual classifications. For this patient, between 14% and 79% of myofibres are misclassified by the frequentist linear regression, compared to the manual classifications. Confusion matrices, shown Table 1, further highlight the differences between the manual and frequentist linear model classifications. All of the misclassifications were false positives, being classified as not-like-control by the frequentist linear model but as like-control by experts.

The problem with the existing frequentist linear model pipeline is highlighted in the classifications: it does not allow single-myofibre OXPHOS protein abundance in patients to deviate from the control subjects’ OXPHOS protein abundance. This assumption is likely too strong, given the natural genetic and environmental variability between human subjects. Ethical and financial constraints typically mean that the number of healthy control subjects from whom biopsies are available is low, usually three or four. The failure of the frequentist linear model for this dataset implies that this is not enough to capture the variability in OXPHOS protein abundance data.

## Methods

### Ethics statement

Data collection and analysis complied with protocols approved by the Ethical Committee of the Martin Luther University Halle-Wittenberg. All subjects provided written informed consent before being included in the study. Two subjects; P07, and P08 were investigated with informed consent by the Newcastle Tyneside Local Research Ethics committees (REC ref. 2002/205). Control tissue was collected with informed consent from patients before undergoing cruciate ligament surgery, with approval from Newcastle and North Tyneside Local Research Ethics Committees (RED ref. 12/NE/0395).

**Table 1:** Frequentist method inconsistently classifies patient myofibres. The confusion matrices when classifying the myofibres of P09, comparing the frequentist linear model’s classifications and the manual classifications. Following Eq 2 a myofibre is labelled as 1 not-like-control and 0 if like-control.

	NDUFB8			CYB			MTCO1		
		Manual			Manual			Manual	
		0	1		0	1		0	1
Freq	0	74	0	0	461	0	0	246	0
	1	451	46	1	79	31	1	281	44

### Bayesian hierarchical model

In healthy control subjects, single myofibre OXPHOS protein abundance appears to have a strong linear relationship to mitochondrial mass [14,15,27]. The same relationship is seen in patient myofibres which are believed healthy by experts. Therefore, we propose that a linear model will be a good fit to the like-control patient and control subject single-myofibre logged OXPHOS protein abundance data.

For data represented in a single 2Dmito plot, we adopt a hierarchical model to explicitly account for inter- and intra-subject variability, as well as the borrowing of strength across different subjects. Moreover, we fit this model in the Bayesian paradigm, which allows the incorporation of prior knowledge for the unknown parameters and coherent propagation of parameter uncertainty in predictions. The model, described below, is independently fitted to the data in one 2Dmito plot. Consequently, for the observed IMC dataset the Bayesian hierarchical model is independently fitted 33 times (11 patients and three OXPHOS proteins). The assumption of independence between data in distinct 2Dmito plots allows generalisation of the model to other observed datasets which could consist of different OXPHOS proteins. Moreover, inclusion of an additional patient level in the hierarchy would require a large number of patients to accurately capture between patient variability.

As with the frequentist linear model, we propose to use the single-myofibre protein abundance data from *k*–1 control subjects. We emphasise that the control data are not aggregated, and we treat each control subject as a separate experimental unit in the hierarchy. The patient forms the final experimental unit, giving *k* groups in total. Each unit contains multiple single-myofibre observations of OXPHOS protein abundance and corresponding mitochondrial mass.

Patient myofibres are classified as part of a two-component mixture model. The first component is intended to model like-control myofibres with the Bayesian hierarchical linear model described above. As it is assumed that all control subject myofibres are healthy, they are modelled using the first component with an associated probability of 1.0. The second component is only fit to patient data and is intended for any myofibre which is not-like-control. The mixture model naturally gives rise to the proportion of not-like-control myofibres, this being equivalent to the probability that an unseen myofibre be modelled with the second component.

However, myofibres showing an OXPHOS defect can display a wide range of protein abundance profiles, see Figs B–D in S1 Appendix. Consequently, adopting a different model for not-like-control myofibres would likely lead to over-fitting. Therefore, we choose the second component to have the same slope and intercept as the first but with a constant, arbitrarily large, variance. The large variance is intended to represent any myofibres whose protein abundance profile is inconsistent with the first component. A constant precision also removes the risk of label switching, a problem commonly seen in Bayesian mixture modelling, see Jasra *et al.* for a discussion [30].

A hierarchical structure is placed on the slope and intercept parameters, giving a different slope and intercept for each control subject and patient, thus allowing for the natural inter-subject variability. Previous work has provided ample support for the correlation between the mitochondrial mass marker VDAC and OXPHOS proteins in healthy controls [14,15,27]. This work also demonstrates a similar relationship in a subset of myofibres from patients and a disruption of this relationship in a proportion of myofibres in patient muscle biopsies, with patient samples consisting of a mix of myofibres that are like-control and not-like-control. Consequently, we assume that the precision in the linear model is equal for all subjects represented in a 2Dmito plot.

The model below describes the *j*^th^ myofibre from subject *i*, from the data represented in a given 2Dmito plot. The log transformed OXPHOS protein abundance is denoted $Y_{ij}$, and the log transformed abundance of VDAC is denoted $X_{ij}$. The prior specification and corresponding parameters are omitted here and discussed in the next section. All parameters in the model below are considered unknown and to be inferred, except *γ*, which is the precision of the second component and assumed fixed and known. The choice of *γ* and its impact on the model are discussed in Sect 3.2.

Let 1 , *…* , *k* - 1 index the control subjects, and *k* correspond to the patient. For *i* = 1 , *…* , *k* - 1


$$\begin{array}{c} Y_{ij}|m_{i},c_{i},\tau \sim N\left(m_{i}X_{ij}+c_{i},\tau^{-1}\right), \end{array}$$ (3)

where $m_{i}$ and $c_{i}$ are the slope and intercept of the *i*^th^ subject and *τ* is the model precision. The patient subject is modelled using a two-component linear mixture model, of the form


$$\begin{array}{llll} Y_{kj}|m_{k},c_{k},\tau ,\gamma \sim \;(1 & -\pi )\;N(m_{k}X_{kj}+c_{k},\tau^{-1})\; & & \;\\ & +\pi \;N(m_{k}X_{kj}+c_{k},\gamma^{-1}). \end{array}$$ (4)

The latent variable $Z_{j}$ denotes the classification of the *j*^th^ patient myofibre. We assume that


$$\begin{array}{c} Z_{j}|\pi \sim Bern(\pi ), \end{array}$$ (5)

where *Bern* ( *p* )  denotes a Bernoulli distribution with probability of success *p*.

Hierarchical priors are placed on the slope and intercept for each subject, for *i* = 1 , *…* , *k*, giving


$$\begin{array}{llll} m_{i}|\mu_{m},\tau_{m} & \sim TN(\mu_{m},\tau_{m}^{-1},0.1),\; & & \;\\ c_{i}|\mu_{c},\tau_{c} & \sim N(\mu_{c},\tau_{c}^{-1}). \end{array}$$ (6)

Here $TN(\mu ,\sigma^{2},a)$ denotes a left-truncated normal distribution on the range  [ *a* , *∞* ) . We have chosen to truncate the normal distribution as it is assumed that the correlation between mitochondrial mass and protein abundance is positive. The parameters $\mu_{m},\mu_{c},\tau_{m}$ and $\tau_{c}$ are the expected values and the precisions of the slope and intercept terms respectively. Their values are inferred from the dataset, and again we defer discussion of their prior specification until the next section.

### Prior specification

The scale and shape of data differ between proteins and data collection methods. For example, IF experiments give images with higher spatial resolution and higher bit depth compared to IMC. Instead of attempting to construct prior beliefs for each data type, we propose using the control data collected under the same experimental conditions. For a specific OXPHOS protein, a simple linear model is fitted to each control subject data independently, in a frequentist setting, to gain a set of estimates of the model parameters; slope, intercept, and precision. The resulting set of parameter estimates is used to inform our beliefs *a priori* by letting the mean of each set be the expected value of $\mu_{m},\mu_{c}$ and *τ*, respectively.

The analysis aims to classify patient myofibres as being like-control or not-like-control in comparison to a set of known healthy myofibres. Using control subject data in the construction of a prior specification, before formal inference, allows us to glean as much information from the control subjects as possible. The choice of prior parameters which are not informed by the control subject data is discussed below. The effect of these parameter uncertainties on the resulting myofibre classifications are assessed in Sect 3.2.

We use normal distributions to summarise our prior beliefs about $\mu_{m}$ and $\mu_{c}$, that is $\mu_{m}\sim N\left(a_{m},b_{m}\right)$ and $\mu_{c}\sim N\left(a_{c},b_{c}\right)$. The values $a_{m}$ and $a_{c}$ are the means of the appropriate frequentist estimates from the control data. As their expectations were informed from the control subject data, we chose prior variances which reflect our fairly high certainty in their values and set $b_{m}=b_{c}=0.25^{2}$.

We use a gamma distribution to summarise our prior beliefs about model precision, so that τ~Ga (g,h), where *g* and *h* denote the shape and rate. The parameters are chosen so that the prior mode matches the mean of the model precisions obtained from the initial fit to the control subject data and the variance is 10.0, representing relatively weak beliefs about precision uncertainty.

The parameters $\tau_{m}$ and $\tau_{c}$ are both summarised by independent gamma distributions, $\tau_{m}\sim Ga\left(g_{m},h_{m}\right)$ and $\tau_{c}\sim Ga\left(g_{c},h_{c}\right)$, *a priori*. Given the variation between samples, it is reasonable to expect their values to be below 100.0 and greater than 1.0. We, therefore, chose a prior mean and variance of 50.0; the shape and rate are then found to be $g_{m}=g_{c}=1.020$ and $h_{m}=h_{c}=51.981$.

Experts have prior beliefs about the proportion of not-like-control myofibres, *π*, however, these beliefs depend heavily on the patient mutation type, age, and the OXPHOS protein in question. For this particular experiment, it is believed that no sample has a not-like-control proportion above 50% in any protein channel [9]. To maintain some generality to analyse all patients with the same prior, we chose a flat prior distribution on the interval  [ 0 . 0 . 5 ] , described by *π* ~ *Uniform* ( 0 , 0 . 5 ) .

The remaining parameter *γ* is not inferred. We choose *γ* to be four or five orders of magnitude smaller than *τ* and set *γ* = 0 . 0001. The impact of different values of *γ* on the resulting myofibre classifications is considered in Sect 3.2.

Fig A in S1 Appendix is a directed acyclic graph showing, for example, dependencies for this model (which model parameters are informed by the control subject data) and highlights the hierarchical structure of the model.

### Computational methods

Inference for this model was carried out using STAN [31] via the R package rstan [32]. STAN offers an efficient exploration of the parameter space and a high effective sample size when compared to JAGS [33], another tool for Bayesian inference. High density intervals (HDIs), referred to throughout, were calculated using the R package HDInterval [34]. Posterior chains were checked for convergence by inspecting the multivariate and individual effective sample sizes for each parameter chain and the R-hat and by visual inspection of trace plots [35,36]. Chains which showed signs of non-convergence were removed. To maintain a constant number of posterior draws, the chain with the highest minimum effective sample size for an individual parameter was used as the basis for inference.

The Bayesian hierarchical model is more computationally intensive than the frequentist linear model, which can classify all myofibres from an entire dataset within a few seconds. However, we believe the computational cost of the model does not hinder its use and the increase in predictive power outweighs any computational cost. In this dataset, there are a total of 1,155 control myofibres and an additional 152–1,199 patient myofibres. This gives between 1,307 to 2,354 data points per 2Dmito plot and per inference scheme. The wall clock time to execute one run of the inference scheme (one chain) for a single patient (and control subjects) ranged between 44 and 155 seconds. The inference scheme was run with 22,000 iterations (including a 20,000 burn-in period), which we find to be satisfactory for convergence. The code was run on a 2023 Macbook Pro with an M2 Pro chip and 16 GB of RAM.

We created an R package, analysis2Dmito, to allow the pipeline and model described here to be used by others. It is available on GitHub, (https://github.com/jordanbchilds/analysis2Dmito) and provides functions to fit the model and visualise the model output, along with complete function documentation, example scripts and guides. The package README.md file on the repository homepage describes how to wrangle data into an appropriate format, for use in the main inference and plotting functions.

Observed and synthetic OXPHOS datasets and relevant R scripts specific to this work can be found in a dedicated GitHub repository, (https://github.com/jordanbchilds/oxphosDeficientClassification). R scripts for analysis are also available in the repository, along with a description of their output in the README file.

## Results

### Model output

**Fig 4:**
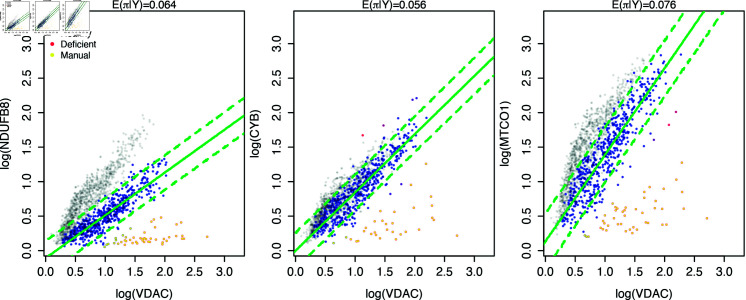
Bayesian model correctly identifies the majority of like-control patient myofibres. Model posterior and classifications for three OXPHOS proteins for P09 with 571 myofibres (coloured points). Control myofibres are shown in grey (1,155 myofibres from four healthy subjects). Patient myofibres are shown on a scale of blue to red, depending on their probability of being not-like-control. The 95% posterior predictive interval and fitted values for the healthy patient (log) OXPHOS abundance and shown in green.

Using the output of the Bayesian hierarchical model, single-myofibre classifications and 95% posterior predictive intervals for patient P09 are shown in Fig 4. All classifications can be seen in Figs B–D in S1 Appendix. Visually, the Bayesian model has performed much better than the frequentist model. In particular, it has not arbitrarily bisected the patient’s like-control myofibre population, unlike the frequentist linear model. The confusion matrices, Table 2, confirm this finding with the percentage of myofibre misclassifications ranging from 0.5% to 1.8%, for this patient. Hence at a single-myofibre level, the Bayesian model classifications are more comparable to the manual classifications by experts than the frequentist linear model.

**Table 2:** Bayesian classification method consistently matches manual classification. Confusion matrices when classifying P09 patient myofibres, comparing the Bayesian classification method to the manual classifications. A single-myofibre was characterised as like-control if the expected marginal posterior probability of the myofibre being not-like-control was less than 50%.

	NDUFB8			CYB			MTCO1		
		Manual			Manual			Manual	
		0	1		0	1		0	1
Bayes	0	525	10	0	525	3	0	539	2
	1	0	36	1	2	41	1	1	29

**Fig 5:**
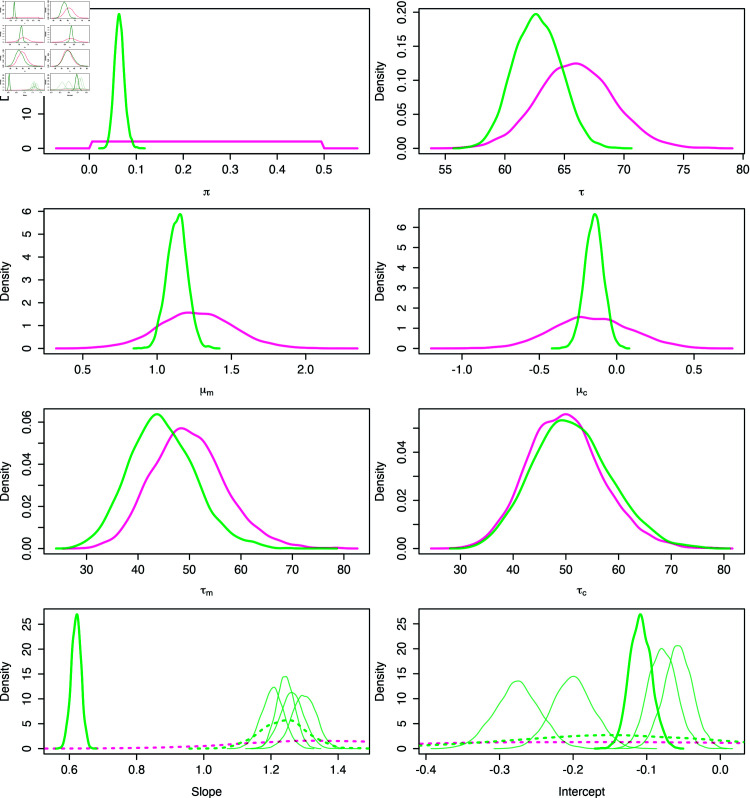
Marginal prior and posterior densities for all parameters after classifying myofibres from P09 by NDUFB8. Kernel density estimates of 20,000 draws from prior (pink) and posterior (green) distributions. Posterior densities of patient slope and intercept are thick, solid green. The control posteriors are shown as transparent green. Dotted lines indicate the population marginal densities of the population-level distributions of the slope and intercept, $\text{N}\left(\mu_{m},\tau_{m}^{-1}\right)$ and $\text{N}\left(\mu_{c},\tau_{c}^{-1}\right)$.

Updated parameter beliefs are encoded by the joint posterior distribution over all levels of the hierarchy, for all datasets represented by a 2Dmito plot. In some cases, little is learnt about the parameters $\tau_{m}$ and $\tau_{c}$, and their marginal posterior distributions closely resemble the prior specification. However, the marginal posterior distributions for the expected slope and intercept parameters, $\mu_{m}$ and $\mu_{c}$, show that the analysis has been informative for all cases. Fig 5 shows the prior specification and marginal posterior distributions for the OXPHOS protein NDUFB8 in P09, shown in the left panel of Fig 4. In this dataset, the parameters $\mu_{m}$ and $\mu_{c}$ do not have substantially different posterior expectations versus the prior equivalent. However, this is to be expected given the high degree of certainty assigned to their *a priori* values. The analysis is particularly informative about the population slope and intercept, as seen in the dashed lines of Fig 5. The reduction in variance seen here is primarily due to the reduction in variance of their expected values, $\mu_{m}$ and $\mu_{c}$. The slopes and intercepts for individual samples in the 2Dmito plot can also be seen in the bottom panels of Fig 5. The variables *π* and *τ* also show a decrease in uncertainty, as seen in Fig 5.

Inspecting the marginal posterior distributions for the slopes and intercepts highlights the inter-subject variability. Fig 5 shows an example of these posteriors, when fitting the Bayesian model to data represented in one 2Dmito plot. Substantial differences can be seen between posterior beliefs of each subject. In this example, the patient slope is much lower than the control subjects, with no overlap in the bulk of their densities. We see a clear difference in the posterior distribution for the model intercept between control subjects. Similar differences can be found throughout the whole observed IMC dataset.

### Sensitivity to prior specification

The inference pipeline requires the prior specification of several parameters, see Fig A in S1 Appendix. A number of prior parameters are informed by control subject data, however, several have been selected by us. To examine the impact, and assess the robustness of the analysis to the prior parameter choices, the inference scheme was implemented with different hyper-parameters. We first consider different values of *γ*; recall that this parameter is not inferred and it is sensible to assume this may impact the resulting posterior distribution and, importantly, myofibre classifications.

**Fig 6:**
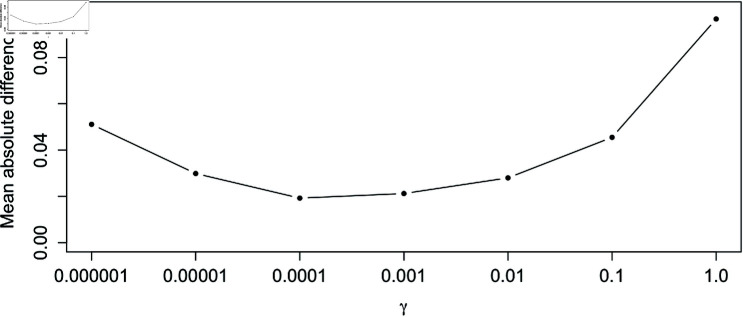
Difference between Bayesian model and manual classification is minimised at *γ* = 0 . 0001. The mean absolute difference (MAD) between the Bayesian proportion of difference and the manual classification calculated across all samples and proteins within the dataset was calculated for varying values of *γ*.

We consider the mean absolute difference (MAD) between the proportion of not-like-control myofibres predicted by the Bayesian model and the proportion found by manual classification for varying values of *γ*. Fig 6 shows that, for the values tested, *γ* = 0 . 0001 minimises this criterion. However, values of *γ* between  [ 0 . 00001 , 0 . 01 ]  resulted in similarly low MADs, indicating that any of these may also be appropriate.

To investigate sensitivity to prior distributions, the model was refit with two sets of priors, one with increased and one with decreased prior uncertainty. We eschew an assessment of the prior uncertainty in *π* as it has a flat prior, as discussed in Sect 2.3. However, the prior uncertainty for all other parameters, $\mu_{m}$, $\tau_{m}$, $\mu_{c}$, $\tau_{c}$ and *τ*, was altered. The prior densities of the altered parameters can be seen in Fig F in S1 Appendix, the prior variances were inflated and deflated by a factor of 5.0.

The two inference schemes are inspected by their inferred value of *π*, see Fig G in S1 Appendix. When comparing the two posterior beliefs, no evidence of a substantial difference between them was found for any 2Dmito plot. A difference was considered substantial if 0.0 lay outside of the posterior 95% high density interval (HDI).

### Comparison with frequentist classifications

**Fig 7:**
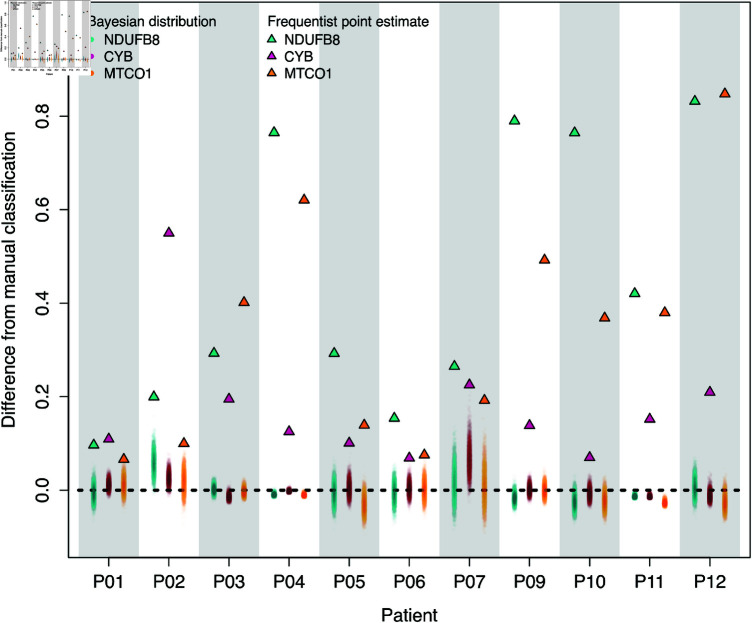
The difference between the frequentist and manual estimates of the proportion of not-like-control is larger than that of the Bayesian and manual estimates. The differences between the manual and frequentist classifications are point estimates and are shown as triangles. The difference between the Bayesian and manual classifications is distributions summarised by 5,000 posterior draws. Each posterior sample of the difference distribution is shown as a small transparent circle. The dashed line is zero. Therefore, the distance between the dashed line and the points is the difference in the estimated proportion of not-like control myofibres from the two methods models and the manual classification.

The Bayesian approach has resulted in a very different classification to the frequentist approach. The differences are highlighted in the proportions of myofibres classified as not-like-control by the two models. They can be seen in Fig 7, which compares the proportion of not-like-control myofibres from the two models to that of the manual classification. The differences between proportions for the frequentist model range between 7% and 85%, with a mean difference of 32%, while the expected differences under the Bayesian model range from –3% to 6%, with a mean expected posterior difference of –0.1%. Here, the expected value was calculated as the posterior median. Further, we calculate the probability of observing the frequentist linear model estimate of the not-like-control proportion or more extreme, given the appropriate Bayesian posterior. The probabilities range is $\left(\leq 2.0\times 10^{-4},6.0\times 10^{-4}\right]$, suggesting a substantial difference between the estimates of the two models for all inferred proportions.

### Simulation study

#### Generating synthetic data.

We investigate the performance of the Bayesian hierarchical model using two synthetic datasets. The first, which we refer to as D01, is a dataset whose OXPHOS abundances resemble that of the observed IMC dataset. The second, D02, has larger OXPHOS abundances to resemble data collected by IF, which have larger protein abundance values due to higher resolution images [14,29]. We also impose a larger inter-subject variability in D02 to demonstrate the flexibility of the Bayesian model.

Synthetic (log) OXPHOS protein abundances were generated for the control subjects and patient represented in each 2Dmito plot from the observed IMC dataset. To generate D01, ground truth parameter values were randomly sampled from the posterior distributions after fitting the Bayesian model to the data represented in each 2Dmito plot. The ground truth OXPHOS status of each synthetic patient myofibre was independently sampled, dependent on the ground truth proportion of not-like-control myofibres. Finally, conditioning on ground-truth parameter values and (log) mitochondrial mass, synthetic (log) OXPHOS protein abundances for like-control myofibres were sampled from component one of the hierarchical model. For not-like-control patient myofibres synthetic (log) OXPHOS protein abundances were sampled from a linear model with lower expectation and higher variance, relative to the like-control protein abundance sampling distribution, to resemble observed under-expressed OXPHOS abundance. To generate D02, (log) mitochondrial mass measurements were linearly transformed and inter-subject variability was increased; the remaining steps were as above. A detailed description of how both synthetic datasets were generated can be found in Text A in S1 Appendix.

#### Model performance on simulated data.

The Bayesian hierarchical model and the frequentist linear model were fitted to the data represented in all synthetic 2Dmito plots. To evaluate model performance we compare two metrics; the difference in posterior proportion of not-like-control myofibres and the classifications of individual myofibres to the ground truth.

After fitting the Bayesian model to D01, a total of six synthetic myofibres, from a possible 19,533, were misclassified when compared to their ground-truth states. For simplicity, the Bayesian model single-myofibre classifications were characterised by their expected marginal posterior probabilities of being not-like-control. If the expected probability was above 50%, the myofibre was characterised as not-like-control and like-control otherwise. Fig L in S1 Appendix shows the 2Dmito plots of the synthetic data which contain the misclassifications. Five of the six misclassified myofibres were false-negatives, being classified as like-control in contradiction to their ground-truth. For all five false-negative classifications, the single-myofibre OXPHOS protein abundances closely resemble that of the like-control myofibres. The one false-positive classification was for an unusually high protein abundance when compared to the rest of the data and it lay outside the 95% posterior predictive interval for like-control OXPHOS protein abundance. Synthetic myofibres showing abnormally high or low abundances are expected when randomly generating large numbers of synthetic data points and thus some misclassifications are to be expected.

The posterior 99% HDIs for *π* contain the ground-truth value for all patients and OXPHOS proteins, showing no evidence of a difference. The range of the expected posterior differences between the Bayesian hierarchical model proportion of not-like-control and ground-truth is  [ - 0 . 07 , 0 . 03 ] . In contrast, the range of the differences between the frequentist model estimate of the not-like-control proportion and the ground-truth is  [ - 0 . 006 , 0 . 868 ] . The posterior probability of observing the frequentist linear model estimates of not-like-control proportion or more extreme, given the Bayesian posterior, is less than 1% for 24 out of 33 estimates in D01. This suggests substantial differences between the classifications of the two models. The difference between the ground-truth not-like-control proportion and those learnt from the two models can be seen in Fig K in S1 Appendix.

For D02, the Bayesian model shows a better fit to the ground-truth than the frequentist linear model, with an expected error in not-like-control proportion between  [ - 0 . 036 , 0 . 055 ]  compared to  [ - 0 . 042 , 0 . 881 ]  under the frequentist model. Again, all ground-truth not-like-control proportions are found within the corresponding posterior 99% HDI, see Fig N in S1 Appendix. As with D01, the model results in some false-negative misclassifications for synthetic myofibres which have abnormally high OXPHOS abundances, see Fig O in S1 Appendix.

Finally, we examine the susceptibility of the Bayesian model to over-fitting by randomly splitting D01 into training and validation subsets, with an 80-20 split. The Bayesian hierarchical model parameters were inferred using only the training data, and the marginal posterior probability of being not-like-control was calculated for all synthetic single-myofibres. These posterior probabilities were compared to those found when inferring parameters on the whole dataset. No evidence of a difference between them was found, by checking that 0.0 lay within the posterior difference 99% HDI, underlining the predictive power of the model.

## Discussion

The proportion of not-like-control myofibres can be an important tool for quantifying the pathological progression of disease over time in skeletal muscle, as well as the effect of disease treatments by comparing the proportions of not-like-control myofibres before and after treatment. Being able to robustly identify like-control (assumed to be healthy) and not-like-control (assumed to have some OXPHOS defect) myofibres within the same individual allows for their direct comparison and to learn about other differences associated with dysfunction.

We have proposed a Bayesian hierarchical model to classify single-myofibres as having mitochondrial dysfunction based on their OXPHOS protein abundance. The model accounts for inter-subject variability by implementing a hierarchical model, borrowing strength between control and patient subjects. In contrast, the frequentist linear model’s binary classifications, the Bayesian inference scheme infers a posterior probability of each patient myofibre being not-like-control, integrating over parameter uncertainty.

The Bayesian model has classifications in agreement with experts’ beliefs compared to the existing frequentist linear model. It has been shown to identify groups of like-control patient myofibres, which the frequentist model misclassified. Additionally, the posterior beliefs of the Bayesian model for both single-myofibre classifications and, consequently, not-like-control proportions show substantially higher agreement with expert manual classification. Allowing for a more robust classification pipeline, which does not rely on the more subjective manual classifications.

When assessing the performance of the two models on synthetic datasets, the Bayesian model outperformed the frequentist linear model, showing better agreement with the ground-truth OXPHOS status of single-myofibres and not-like-control myofibre proportions. We also found that the Bayesian model performed well when the synthetic data was split into training and validation subsets, showing no evidence of different myofibre classifications compared to fitting the model to the whole dataset.

We use a prior construction pipeline which allows for a prior to be informed by previous experiments, or, as used here, control subject data. Prior sensitivity was explored by inflating and deflating their prior uncertainty, without including unrealistic areas of the support. These findings suggest that the results obtained are relatively insensitive to the prior specification. The prior specification was also implemented on the synthetic datasets, showing different OXPHOS profiles and scales, where the model was able to retrieve ground-truth parameter values and single-myofibre classifications.

The model can be fitted by our R package, analysis2Dmito, available on GitHub (https://github.com/jordanbchilds/analysis2Dmito). The package allows the user to fit the model, executing the Bayesian inference via STAN [31], and automatically constructs prior distributions based upon the pipeline shown here. If desired, the user can specify prior parameters; however, the choice of prior distributions cannot be changed. Given the extensive range of prior beliefs that can be constructed using the distributions implemented, we believe this is not impactful.

The model has limitations; it requires healthy patient myofibres to show a linear relationship in log OXPHOS protein abundance and log mitochondrial mass, similar to the controls, and be distinct from OXPHOS abundances from not-like-control myofibres. Due to the large and fixed precision of the second component in the Bayesian model, the model can successfully work on a range of not-like-control proportions and mutations. However, an overwhelming number of not-like-control myofibres would increase the likelihood of the linear regression fitting to the not-like-control population, resulting in misclassification. As such, this classification approach is unreliable for patients with more than 50% not-like-control myofibres. In a diagnostic context, the proportion of affected myofibres cannot be known *a priori*. Nevertheless, for patients with fewer than 50% of not-like-control myofibres, our approach greatly outperforms the current frequentist approach.

Here, we developed a Bayesian hierarchical approach to classify myofibres as like-control or not-like-control with respect to their OXPHOS protein abundance and demonstrated that it is comparable to expert manual classification. The Bayesian hierarchical model allows for the natural variation between subjects and significantly improves previous methods.

## Supporting information

S1 Appendix Text A. Description of synthetic data generation. **Fig A**. Directed acyclic graph of the Bayesian hierarchical linear regression model. Inferred parameters are shown in circles, and known parameters are shown within boxes. Boxes highlighted in blue are informed by the control data, and the boxes highlighted in yellow are chosen by us. The functions *m* ( ⋅ )  and *v* ( ⋅ )  indicate the mode and variance for a parameter. The dotted boxes enclosing parameters indicate which parameters occur for every sample (the outer box) and which occur for every myofibre in a sample (the inner box). For simplicity, a new variable is introduced, ${\hat{\tau }}_{ij}$, the precision used to model the *j*^th^ myofibre in the *i*^th^ sample. It is defined as *τ*, if *i* is a control or $Z_{j}=0$, and *γ* otherwise. **Fig B–D**. Posterior myofibre classifications and posterior 95% predictive interval for all OXPHOS proteins and patients. Protein abundances from all control subjects are shown in black. The patient myofibres are coloured on a scale from red to blue, red being not-like-control and blue being healthy, based on their posterior expected marginal probability of being not-like-control. The posterior expected value and 95% predictive interval for the linear model fit to the healthy patient cells are shown as solid and dashed green lines. **Fig E**. Posterior beliefs of the proportion of myofibres with are not-like-controls for all patients and OXPHOS proteins. Twenty thousand draws from their posterior distributions represent the beliefs. **Fig F**. Wide and narrow prior distributions used to inspect the impact of parameter uncertainty. The two sets of prior distributions for all patients and OXPHOS protein NDUFB8, used in Sect 3.2 to assess hyperparameter uncertainty, which we chose and not informed from the control data. **Fig G**. No difference in the not-like-control proportion from models with varying amounts of prior uncertainty. The difference in the *π* posterior between the original priors and the narrow/wider ones. The prior variance for the precisions, $\tau_{m},\tau_{c}$ and *τ*, were increased or decreased by a factor of 5.0 for the wide and narrow priors, respectively. The prior variance for the expected slope and intercept, $\mu_{m}$ and $\mu_{c}$, were increased and decreased by a factor of 5.0. **Fig H**. Synthetic data generated for patient P12, D01. OXPHOS protein abundances for control data are shown by black points and synthetic patient data is shown in coloured points. Synthetic patient myofibres are coloured blue if their ground-truth state is like-control and red with a yellow dot if their ground-truth state is not-like-control. **Fig I**. The Bayesian hierarchical model able to find ground-truth parameter values in synthetic dataset D01. Posterior beliefs and model fit for OXPHOS protein in patient P09, of the synthetic dataset D01. Ground-truth parameter values, used to generate the data, are shown with vertical dotted lines. Prior and posterior beliefs are shown in pink and green respectively for all parameters except the slope and intercept for control subjects. Each subject was given a different colour to distinguish between ground-truth values for each control subject. The 2Dmito plot shows the 95% posterior predictive interval and expected value for the linear model fitted to the patient subject. **Fig J**. The Bayesian hierarchical model more accurately estimates the not-like-control proportion compared to the frequentist model in synthetic data D01. The posterior difference between the ground-truth not-like-control proportion and the Bayesian hierarchy’s beliefs. Also shown are the differences between the frequentist estimates and the ground-truth. Stars indicate the probability of observing the frequentist linear model’s estimate, given the Bayesian posterior, is greater than 1%. **Fig K**. Misclassifications of synthetic data D01 are due to data overlap. Synthetic OXPHOS data and the posterior predictive of the Bayesian hierarchical model. Posterior Bayesian classifications are simplified to be whether the expected marginal posterior probability of being not-like-control is above 0.5. Misclassifications are highlighted with a yellow square or diamond, depending on the misclassification type, see legend. **Fig L**. Synthetic data generate for patient P12, D01. OXPHOS protein abundances for control data are shown by black points and synthetic patient data is shown in coloured points. Synthetic patient myofibres are coloured blue if their ground-truth state is like-control and red with a yellow dot if their ground-truth state is not-like-control. **Fig M**. The Bayesian hierarchical model able to find ground-truth parameter values in synthetic dataset D02. Posterior beliefs and model fit for OXPHOS protein in patient P12, of the synthetic dataset D02. Ground-truth parameter values, used to generate the data, are shown with vertical dotted lines. Prior and posterior beliefs are shown in pink and green respectively for all parameters except the slope and intercept for control subjects. Each subject was given a different colour to distinguish between ground-truth values for each control subject. The 2Dmito plot shows the 95% posterior predictive interval and expected value for the linear model fitted to the patient subject. **Fig N**. The Bayesian hierarchical model more accurately estimates the not-like-control proportion compared to the frequentist model with synthetic data D02. The posterior difference between the ground-truth not-like-control proportion and the Bayesian hierarchy’s beliefs. Also shown are the differences between the frequentist estimates and the ground-truth. Stars indicate the probability of observing the frequentist linear model’s estimate, given the Bayesian posterior, is greater than 1%, showing a lack of significance between the two methods. **Fig O**. Misclassifications of synthetic data D02 are due to data overlap. Synthetic OXPHOS data and the posterior predictive of the Bayesian hierarchical model. Posterior Bayesian classifications are simplified to be whether the posterior expected marginal probability of being not-like-control is above 0.5. Misclassifications are highlighted with a yellow square or diamond, depending on the misclassification type, see legend. (PDF)
