# Supplementary material for: Bayesian classification of OXPHOS deficient skeletal myofibres
Source: PLoS Comput Biol. 2025 Feb 19;21(2):e1012770. doi: 10.1371/journal.pcbi.1012770 (PMC11838899; doi:10.1371/journal.pcbi.1012770)
Supplement: S1 Appendix — Description of synthetic data generation. Fig A. Directed acyclic graph of the Bayesian hierarchical linear regression model. Inferred parameters are shown in circles, and known parameters are shown within boxes. Boxes highlighted in blue are informed by the control data, and the boxes highlighted in yellow are chosen by us. The functions m ( ⋅ ) and v ( ⋅ ) indicate the mode and variance for a parameter. The dotted boxes enclosing parameters indicate which parameters occur for every sample (the outer box) and which occur for every myofibre in a sample (the inner box). For simplicity, a new variable is introduced, τ^ij, the precision used to model the jth myofibre in the ith sample. It is defined as τ, if i is a control or Zj=0, and γ otherwise. Fig B–D. Posterior myofibre classifications and posterior 95% predictive interval for all OXPHOS proteins and patients. Protein abundances from all control subjects are shown in black. The patient myofibres are coloured on a scale from red to blue, red being not-like-control and blue being healthy, based on their posterior expected marginal probability of being not-like-control. The posterior expected value and 95% predictive interval for the linear model fit to the healthy patient cells are shown as solid and dashed green lines. Fig E. Posterior beliefs of the proportion of myofibres with are not-like-controls for all patients and OXPHOS proteins. Twenty thousand draws from their posterior distributions represent the beliefs. Fig F. Wide and narrow prior distributions used to inspect the impact of parameter uncertainty. The two sets of prior distributions for all patients and OXPHOS protein NDUFB8, used in Sect 3.2 to assess hyperparameter uncertainty, which we chose and not informed from the control data. Fig G. No difference in the not-like-control proportion from models with varying amounts of prior uncertainty. The difference in the π posterior between the original priors and the narrow/wider ones. The prior variance fo [file pcbi.1012770.s001.pdf]

Supplementary material

Text A. Generating synthetic data

Here, we give details of the data generation mechanism used to give the synthetic datasets analysed in Section 3.4.

Generating D01

Synthetic OXPHOS protein abundance is created for each OXPHOS protein and patient subject, i.e. synthetic data is generated for each 2Dmito plot in the observed IMC dataset. For a specific OXPHOS protein and patient combination, a set of ground-truth parameter values are generated by randomly sampling from the posterior distributions of the Bayesian hierarchical model fitted to the observed data of that 2Dmito plot. Consequently, control data for each patient will differ. First, we set ground-truth values for all parameters except the slope and intercepts of each experimental unit. This is achieved by independently sampling from their respective marginal posterior distributions.

The remaining parameters, the slopes and intercepts for each experimental unit, are sampled dependently on the previously set ground-truth values. Let $k-1$ be the number of control subjects, then for $i = 1, \ldots, k$, the ground-truth slopes and intercepts are sampled via

$$\begin{aligned} {c_i}^*|{\mu_c}^*, {\tau_c}^* &\sim \mathrm{N}\left({\mu_c}^*, {{\tau_c}^*}^{-1}\right) \\ {m_i}^*|{\mu_m}^*, {\tau_m}^* &\sim \mathrm{N}\left({\mu_m}^*, \frac{1}{{\tau_m}^*}\right) \end{aligned} \tag{1}$$

where * indicates a ground-truth parameter. The latent states, Z, denoting the OXPHOS status of each patient myofibre are sampled from a Bernoulli distribution. Let N_i be the number of myofibres in the i-th experimental unit then for $j = 1, \ldots, N_k$

$$ {Z_j}^*|\pi^* \sim \mathrm{Bern}\left(\pi^*\right). \tag{2}$$

Finally, the synthetic log OXPHOS protein abundance, Y_{ij}, is sampled by conditioning on the log mitochondrial mass, X_{ij}, and appropriate ground-truth parameters. For $i = 1, \ldots, k-1$ and $j = 1, \ldots, N_i$,

$$Y_{ij}^* | m_i^*, c_i^*, \tau^* \sim \mathrm{N}\left(m_i^* X_{ij} + c_i^*, \frac{1}{\tau^*} \right). \tag{3}$$

The synthetic log OXPHOS protein abundance for each patient is sampled conditional on the myofibre's OXPHOS state, Z_j^*, and logged mitochondrial mass, X_{kj}. For not-like-control patient myofibres, the expected logged OXPHOS protein abundance is shifted down, relative to the expected like-control abundance and model error, and the sampling variance is increased. This is intended to resemble the low correlation and low protein abundances seen in not-like-control myofibres. For $j = 1, \ldots, N_k$,

$$\begin{aligned} Y_{kj}^* | m_k^*, c_k^*, \tau^*, Z_j^* = 0 &\sim \mathrm{N}\left(m_k^* X_{kj} + c_k^*, \frac{1}{\tau^*} \right), \\ Y_{kj}^* | m_k^*, c_k^*, \tau^*, Z_j^* = 1 &\sim \mathrm{N}\left(m_k^* X_{kj} + c_k^* - \frac{11}{\sqrt{\tau^*}}, \frac{10}{\tau^*} \right). \end{aligned} \tag{4}$$

Fig S8, show example data from D01.

Generating D02

To create single-myofibre OXPHOS abundance data which is on a similar scale to abundances collected by IF, the the measures of mitochondrial mass are linearly transformed. For this dataset, the ground-truth model precisions are decreased by a factor of 4.0 after being randomly sampled from its posterior distribution. Eq. 3 and 4 become

$$Y_{ij}^* | m_i^*, c_i^*, \tau^* \sim \mathrm{N}\left(\frac{1}{2} m_i^* \left(3X_{ij} + 8\right) + c_i^*, \frac{1}{\tau^*} \right), \tag{5}$$

and

$$\begin{aligned} Y_{kj}^* | m_k^*, c_k^*, \tau^*, Z_j^* = 0 &\sim \mathrm{N}\left(\frac{1}{2} m_k^* \left(3X_{kj} + 8\right) + c_k^*, \frac{1}{\tau^*} \right), \\ Y_{kj}^* | m_k^*, c_k^*, \tau^*, Z_j^* = 1 &\sim \mathrm{N}\left(\frac{1}{2} m_k^* \left(3X_{kj} + 8\right) + c_k^* - \frac{11}{\sqrt{\tau^*}}, \frac{10}{\tau^*} \right). \end{aligned} \tag{6}$$

The increase in (log) mitochondrial mass increases the inter-subject variability. The synthetic dataset generated by this method is denoted D02 and example 2Dmito plots can be seen in Fig S12.

Additional figures

Directed Acyclic Graph

The Directed Acyclic Graph (DAG) for the proposed hierarchical model. The common notation of parameterising a γ distribution by its shape and rate is kept, but we show that the shape and rate are dependent on a mode and variance. For simplicity, a new variable is introduced, $\hat{\tau}_{ij}$, the precision used to model the jth cell in the ith sample. It is defined as

$$\hat{\tau}_{ij} = \begin{cases} \tau, & i \text{ is a control or } Z_j = 0 \\ \gamma, & i \text{ is not control and } Z_j = 1 \end{cases}$$

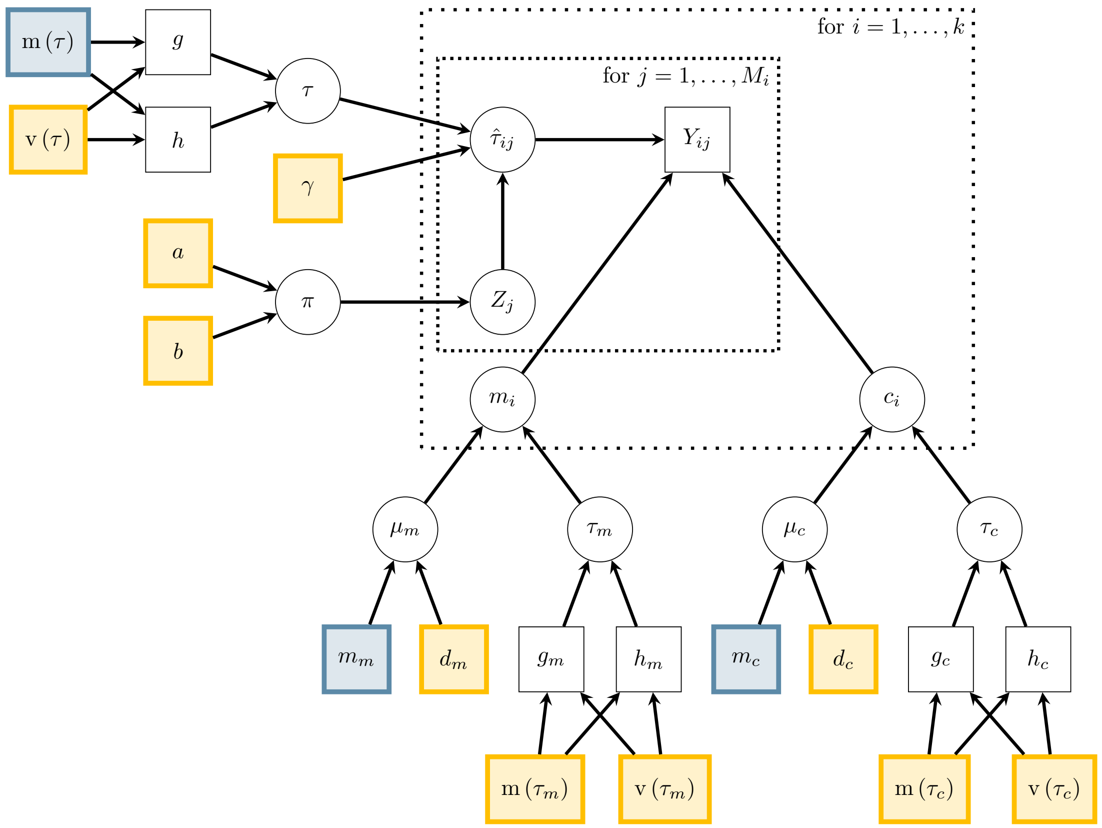

Fig A. Directed acyclic graph of the Bayesian hierarchical linear regression model. Inferred parameters are shown in circles, and known parameters are shown within boxes. Boxes highlighted in blue are informed by the control data, and the boxes highlighted in yellow are chosen by us. The functions m(·) and v(·) indicate the mode and variance for a parameter. The dotted boxes enclosing parameters indicate which parameters occur for every sample (the outer box) and which occur for every myofibre in a sample (the inner box). For simplicity, a new variable is introduced, $\hat{\tau}_{ij}$, the precision used to model the jth myofibre in the ith sample. It is defined as τ, if i is a control or $Z_j = 0$, and γ otherwise.

Output for observed data

The posterior classifications and 95% posterior predictive interval for each patient sample.

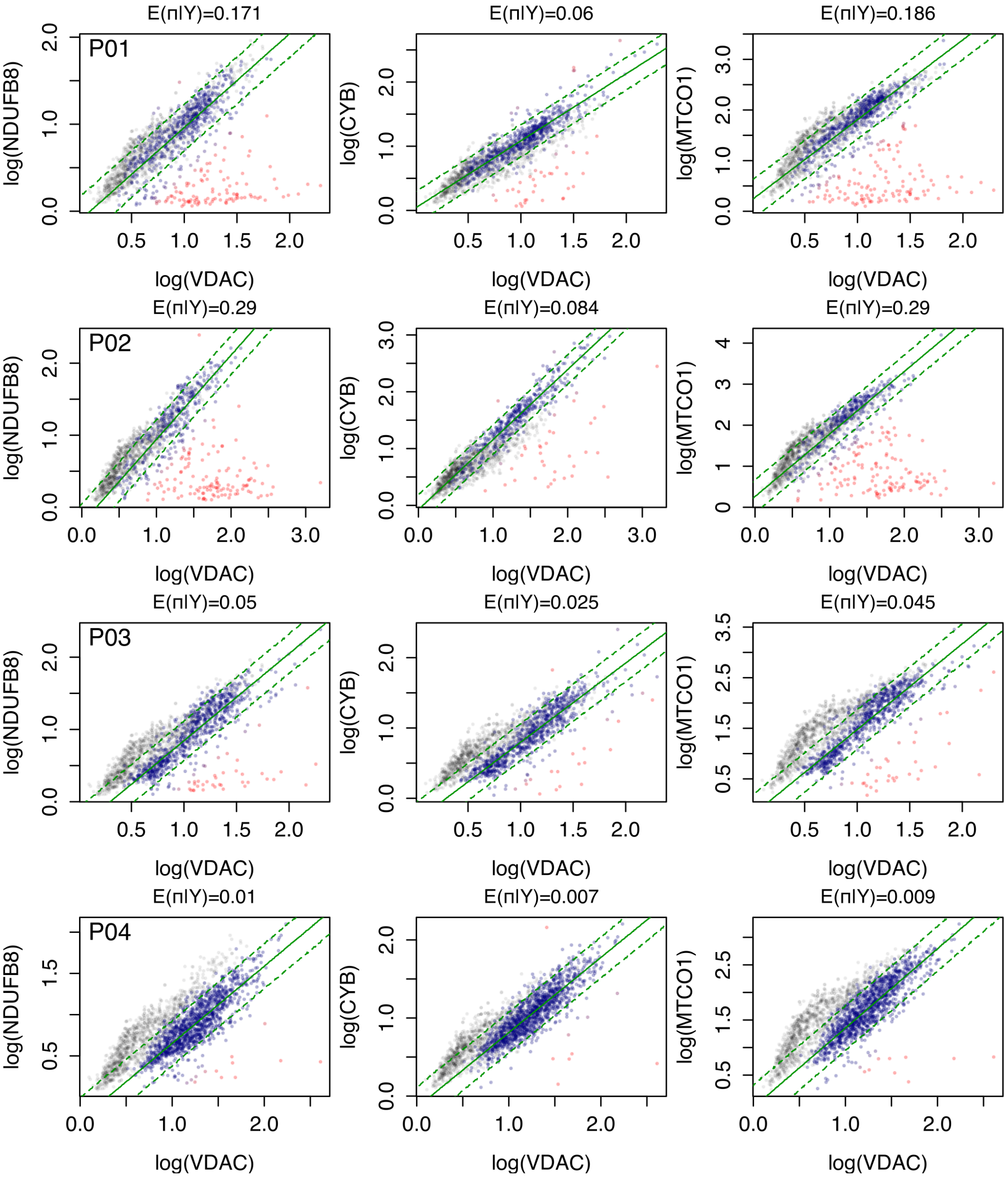

Fig B. Posterior cell classifications and posterior 95% predictive interval for all OXPHOS proteins and patient P01 to P04. Protein abundances from all control subjects are shown in black. The patient cells are coloured on a scale from red to blue, red being not-like-control and blue being healthy, based on their posterior expected marginal probability of being not-like-control. The posterior expected value and 95% predictive interval for the linear model fit to the healthy patient cells are shown as solid and dashed green lines.

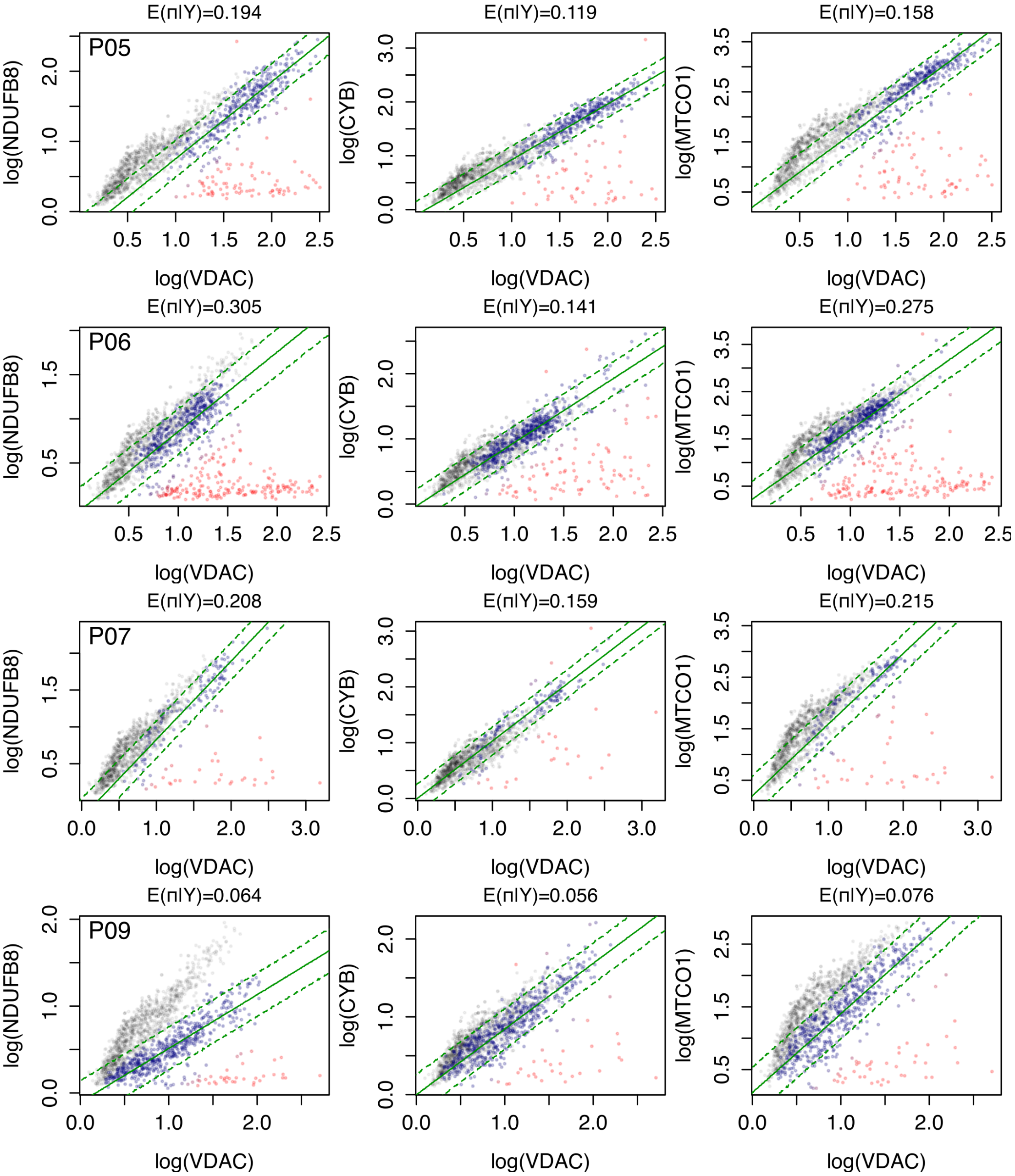

Fig C. Posterior cell classifications and posterior 95% predictive interval for all OXPHOS proteins and patient P05 to P08. Protein abundances from all control subjects are shown in black. The patient cells are coloured on a scale from red to blue, red being not-like-control and blue being healthy, based on their posterior expected marginal probability of being not-like-control. The posterior expected value and 95% predictive interval for the linear model fit to the healthy patient cells are shown as solid and dashed green lines.

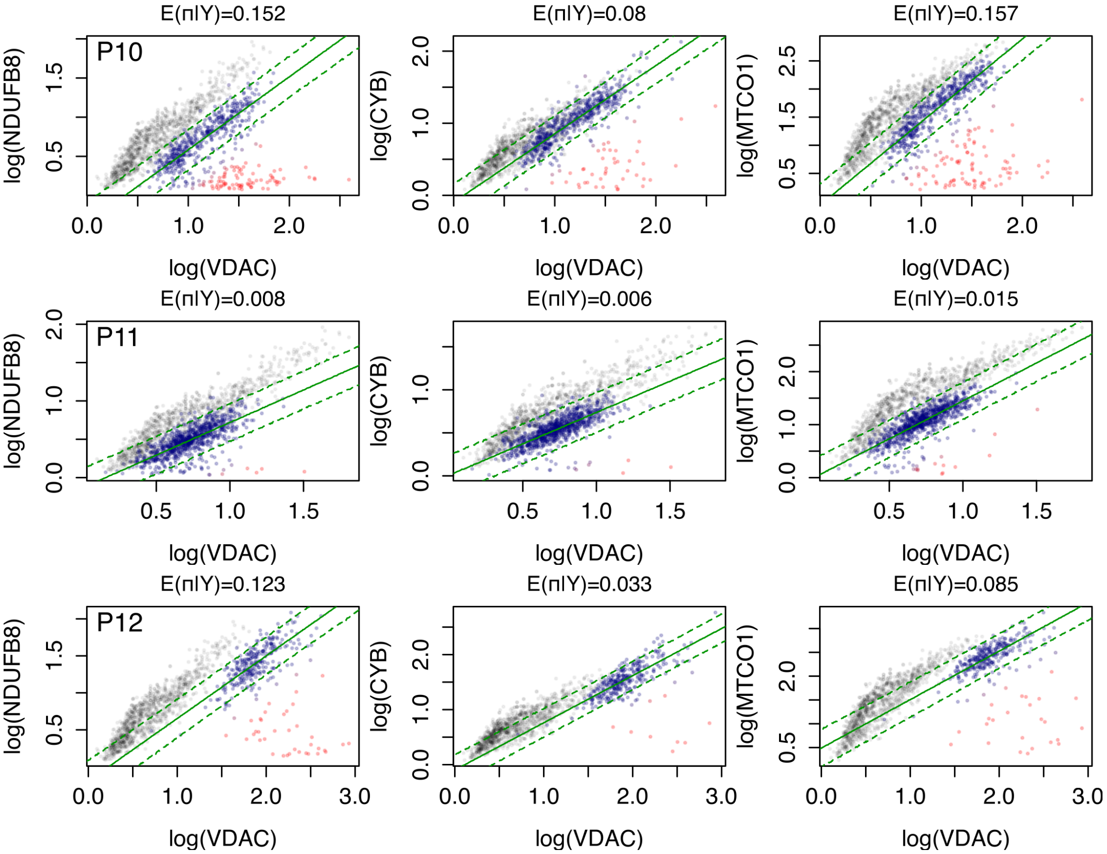

Fig D. Posterior cell classifications and posterior 95% predictive interval for all OXPHOS proteins and patient P09 to P11. Protein abundances from all control subjects are shown in black. The patient cells are coloured on a scale from red to blue, red being not-like-control and blue being healthy, based on their posterior expected marginal probability of being not-like-control. The posterior expected value and 95% predictive interval for the linear model fit to the healthy patient cells are shown as solid and dashed green lines.

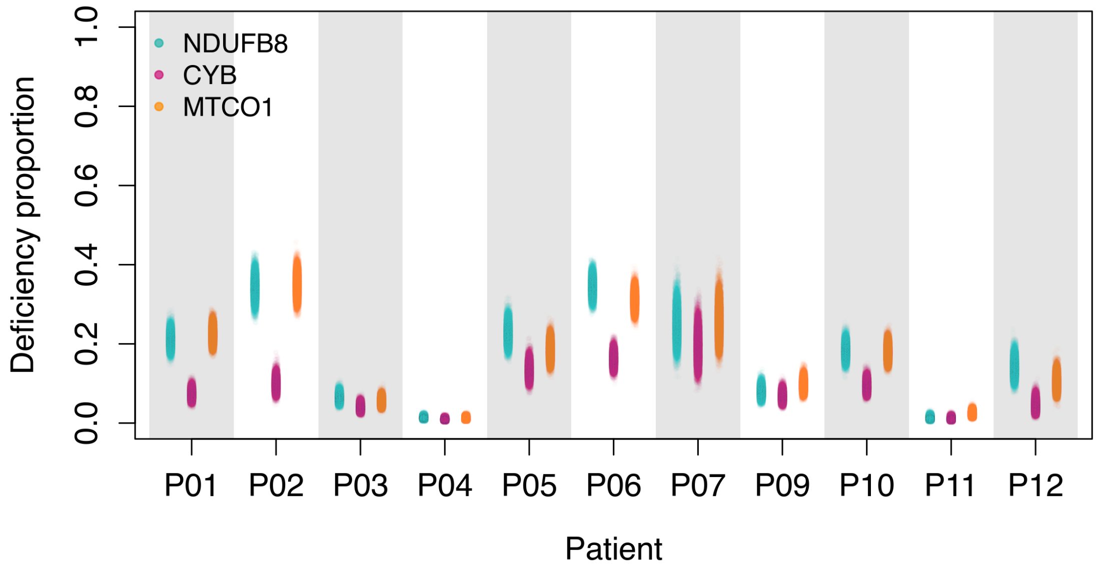

Fig E. Posterior beliefs of the proportion of myofibres with are not-like-controls for all patients and OXPHOS proteins. Twenty thousand draws from their posterior distributions represent the beliefs.

Prior impact

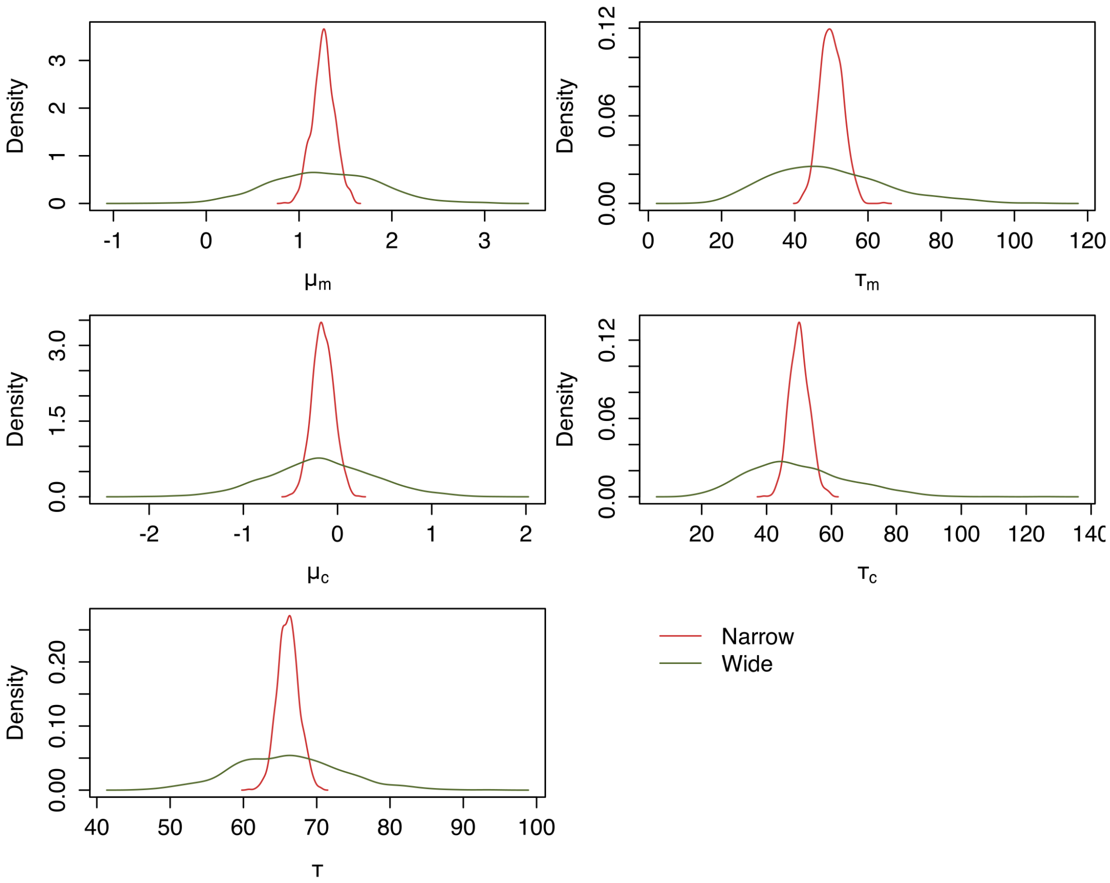

Fig F. Wide and narrow prior distributions used to inspect the impact of parameter uncertainty. The two sets of prior distributions for all patients and OXPHOS protein NDUFB8, used in Section 3.2 to assess hyperparameter uncertainty, which we chose and not informed from the control data.

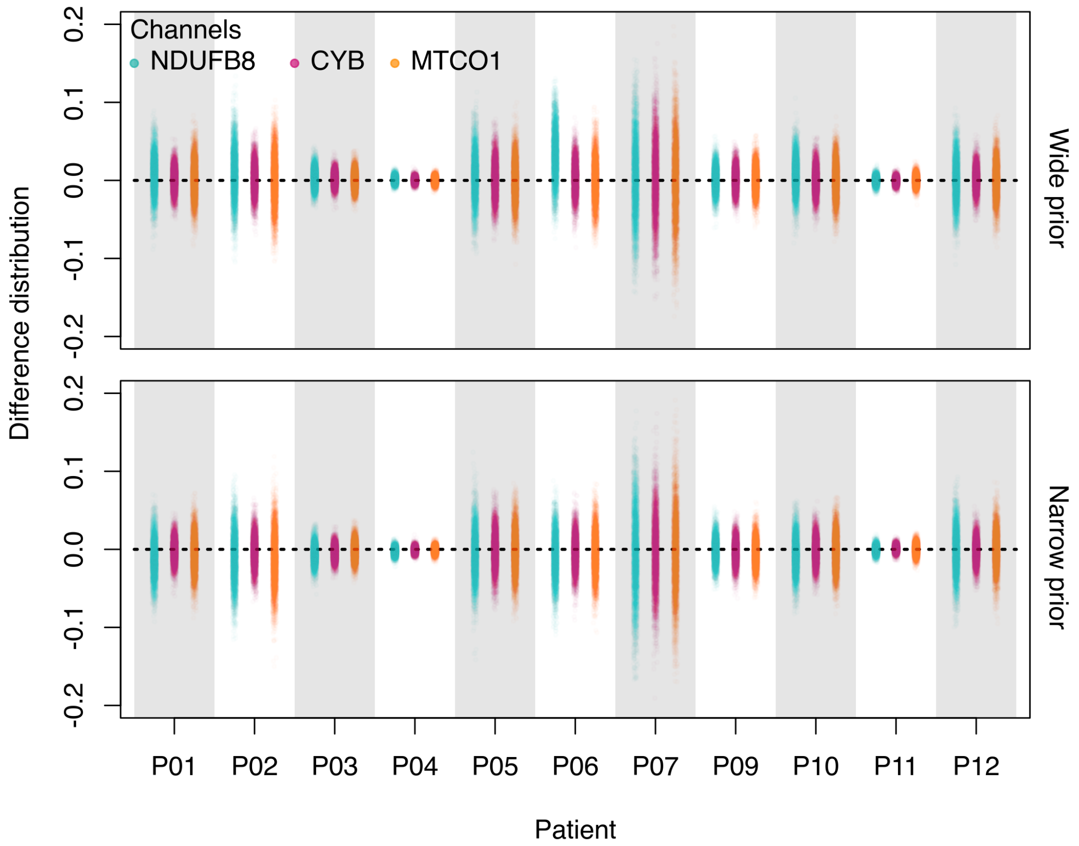

Fig G. No difference in the not-like-control proportion from models with varying amounts of prior uncertainty. The difference in the π posterior between the original priors and the narrow/wider ones. The prior variance for the precisions, τ_m, τ_c and τ, were increased or decreased by a factor of 5.0 for the wide and narrow priors, respectively. The prior variance for the expected slope and intercept, μ_m and μ_c, were increased and decreased by a factor of 5.0.

Output for synthetic data

Synthetic data D01

An example of the synthetic data generated directly from the model is given in Fig S8.

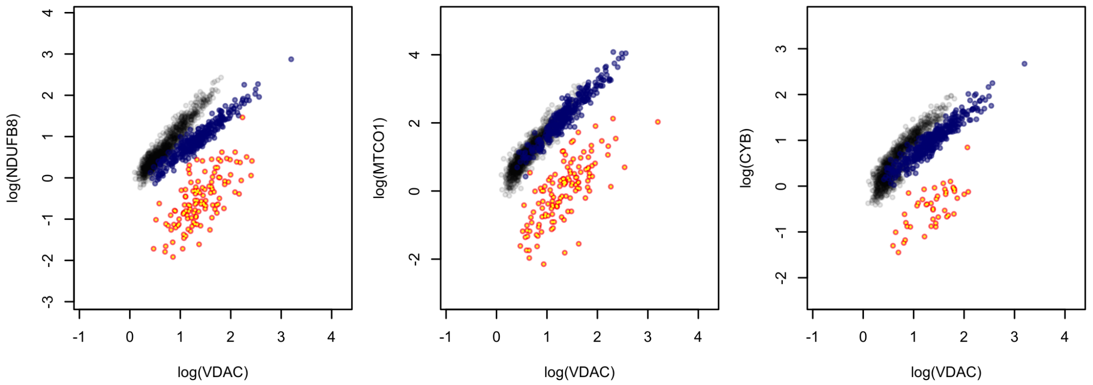

Fig H. Synthetic data generated for patient P12, D01. OXPHOS protein abundances for control data are shown by black points and synthetic patient data is shown in coloured points. Synthetic patient myofibres are coloured blue if their ground-truth state is like-control and red with a yellow dot if their ground-truth state is not-like-control.

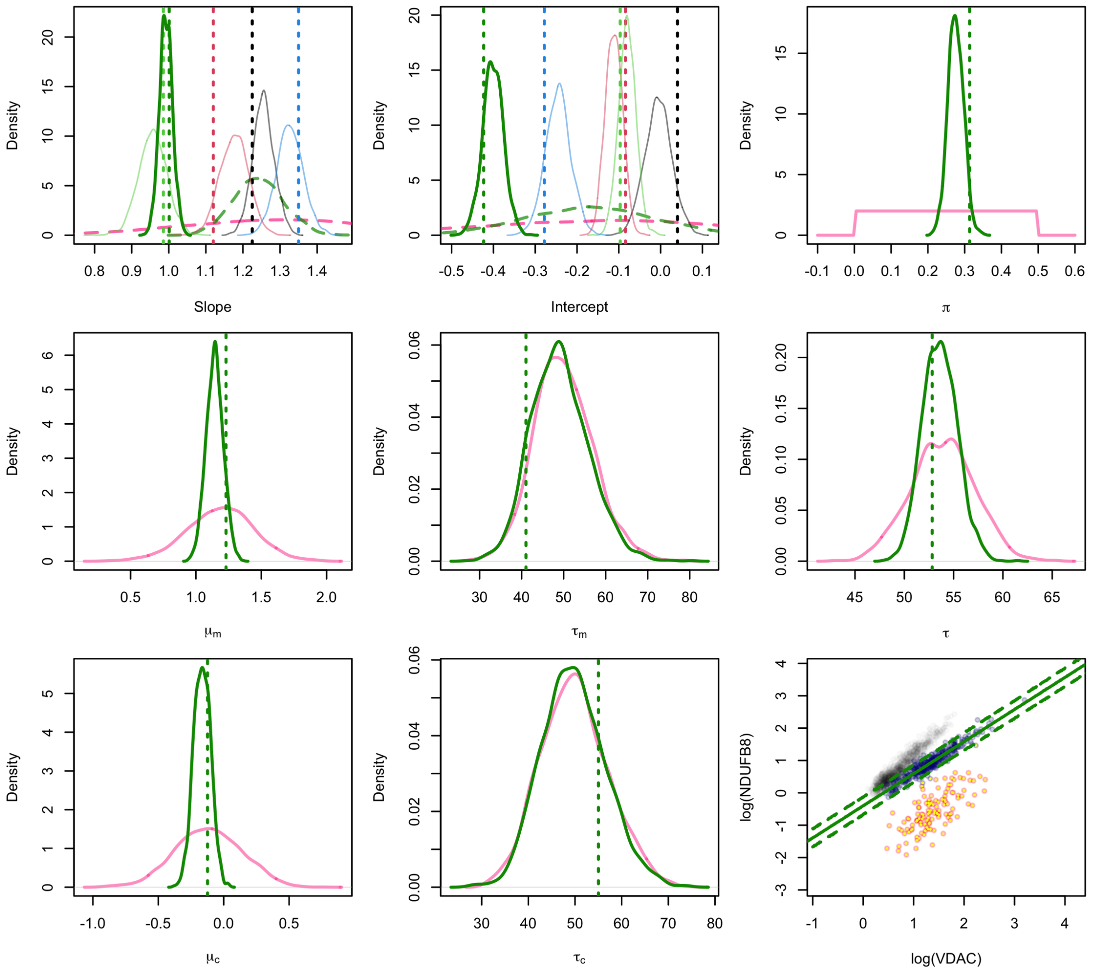

Fig I. The Bayesian hierarchical model able to find ground-truth parameter values in synthetic dataset D01. Posterior beliefs and model fit for OXPHOS protein in patient P09, of the synthetic dataset D01. Ground-truth parameter values, used to generate the data, are shown with vertical dotted lines. Prior and posterior beliefs are shown in pink and green respectively for all parameters except the slope and intercept for control subjects. Each subject was given a different colour to distinguish between ground-truth values for each control subject. The 2Dmito plot shows the 95% posterior predictive interval and expected value for the linear model fitted to the patient subject.

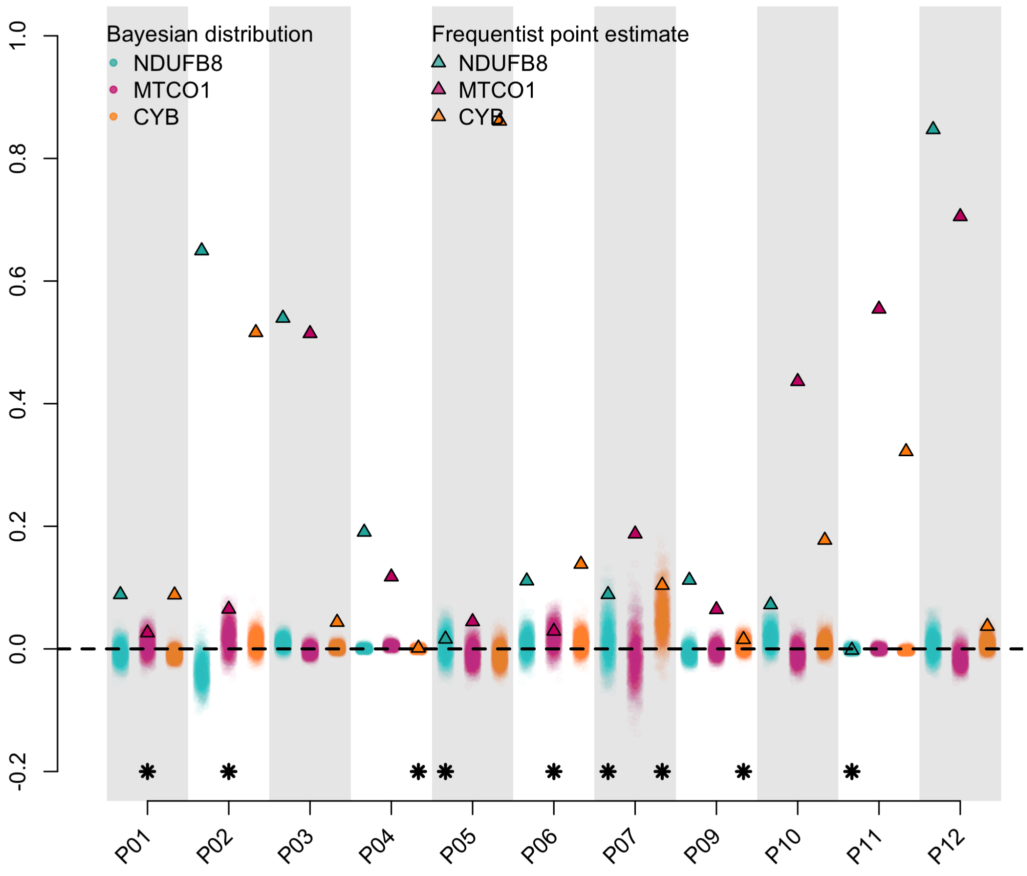

Fig J. The Bayesian hierarchical model more accurately estimates the not-like-control proportion compared to the frequentist model in synthetic data D01. The posterior difference between the ground-truth not-like-control proportion and the Bayesian hierarchy's beliefs. Also shown are the differences between the frequentist estimates and the ground-truth. Stars indicate the probability of observing the frequentist linear model's estimate, given the Bayesian posterior, is greater than 1%.

The misclassifications made by the Bayesian hierarchical model on the synthetic dataset D01 are shown in Fig S11.

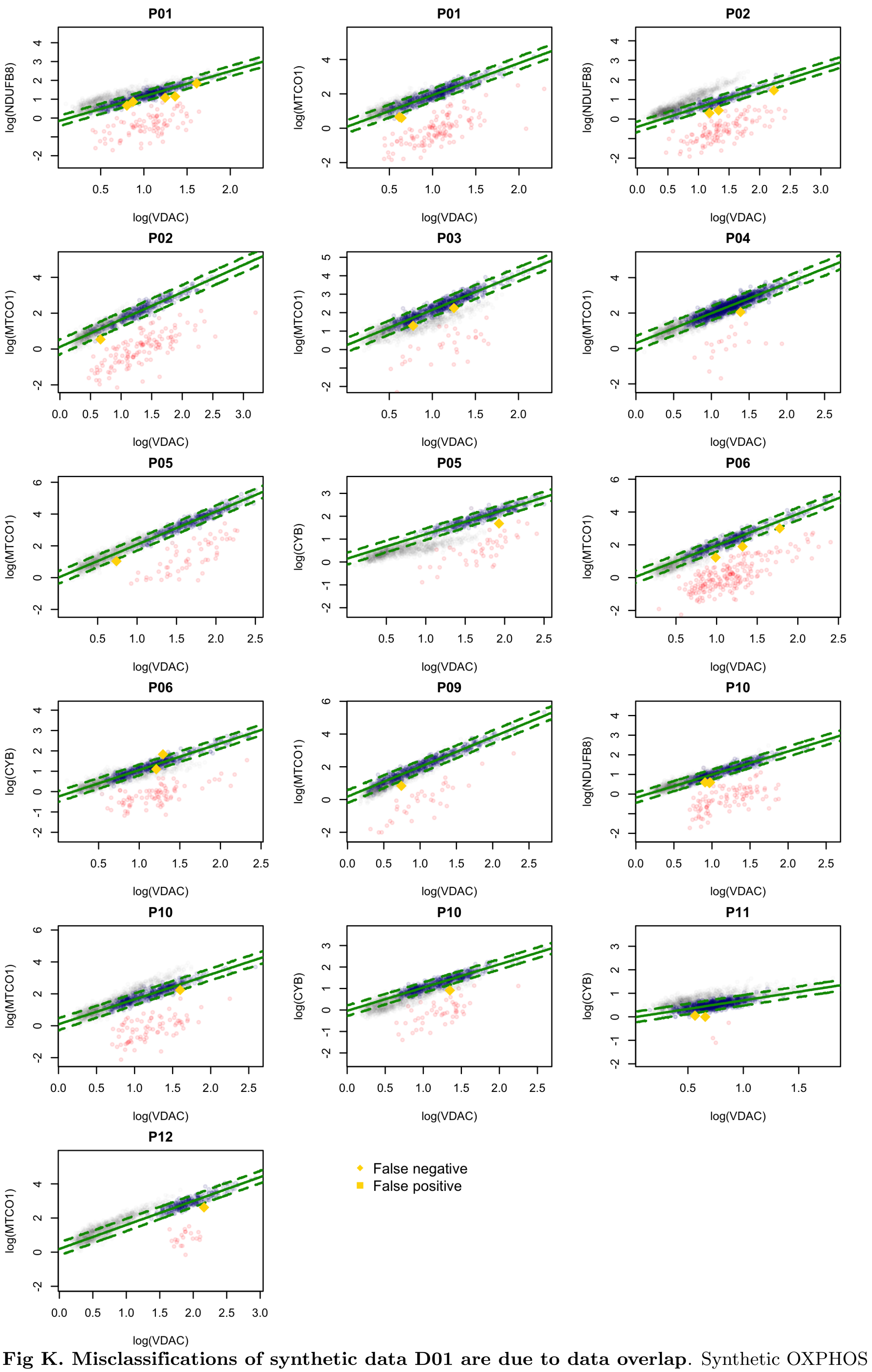

Fig K. Misclassifications of synthetic data D01 are due to data overlap. Synthetic OXPHOS data and the posterior predictive of the Bayesian hierarchical model. Posterior Bayesian classifications are simplified to be whether the expected marginal posterior probability of being not-like-control is above 0.5. Misclassifications are highlighted with a yellow square or diamond, depending on the misclassification type, see legend.

Output for synthetic dataset D02

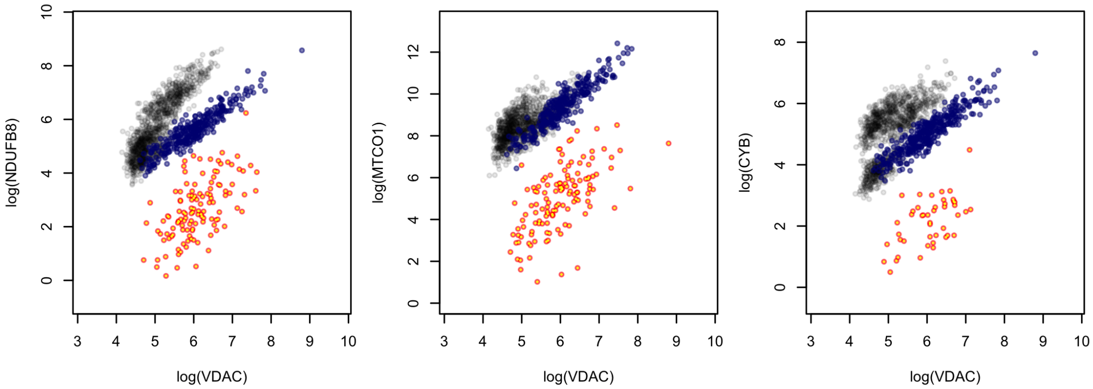

Fig L. Synthetic data generate for patient P12, D01. OXPHOS protein abundances for control data are shown by black points and synthetic patient data is shown in coloured points. Synthetic patient myofibres are coloured blue if their ground-truth state is like-control and red with a yellow dot if their ground-truth state is not-like-control.

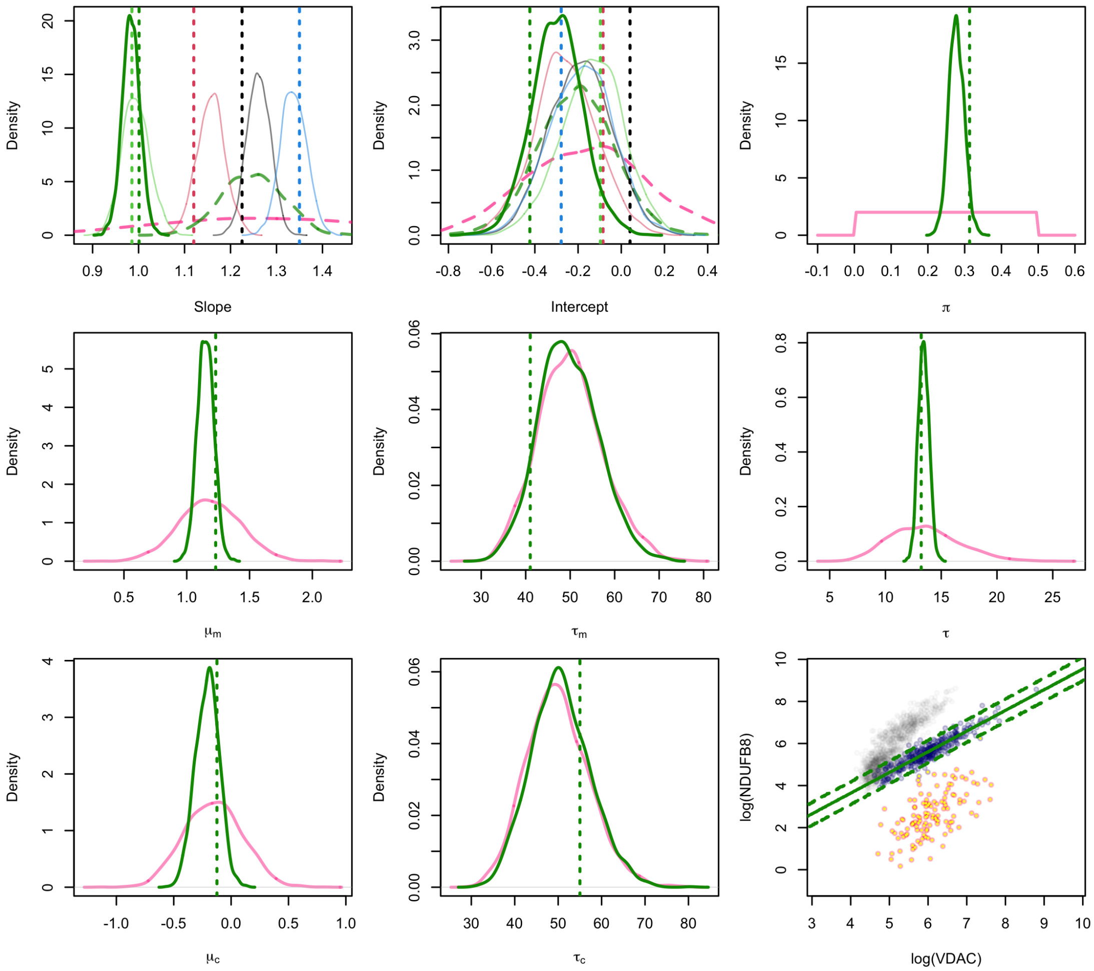

Fig M. The Bayesian hierarchical model able to find ground-truth parameter values in synthetic dataset D02. Posterior beliefs and model fit for OXPHOS protein in patient P12, of the synthetic dataset D02. Ground-truth parameter values, used to generate the data, are shown with vertical dotted lines. Prior and posterior beliefs are shown in pink and green respectively for all parameters except the slope and intercept for control subjects. Each subject was given a different colour to distinguish between ground-truth values for each control subject. The 2Dmito plot shows the 95% posterior predictive interval and expected value for the linear model fitted to the patient subject.

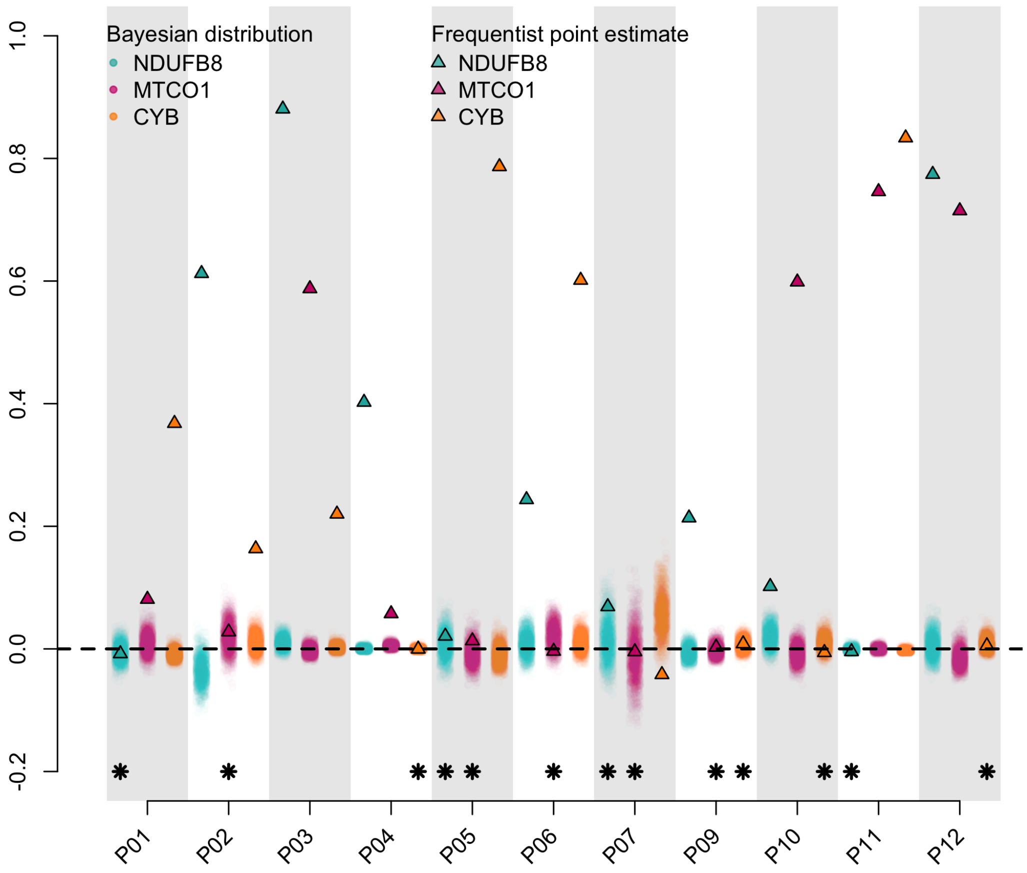

Fig N. The Bayesian hierarchical model more accurately estimates the not-like-control proportion compared to the frequentist model with synthetic data D02. The posterior difference between the ground-truth not-like-control proportion and the Bayesian hierarchy's beliefs. Also shown are the differences between the frequentist estimates and the ground-truth. Stars indicate the probability of observing the frequentist linear model's estimate, given the Bayesian posterior, is greater than 1%, showing a lack of significance between the two methods.

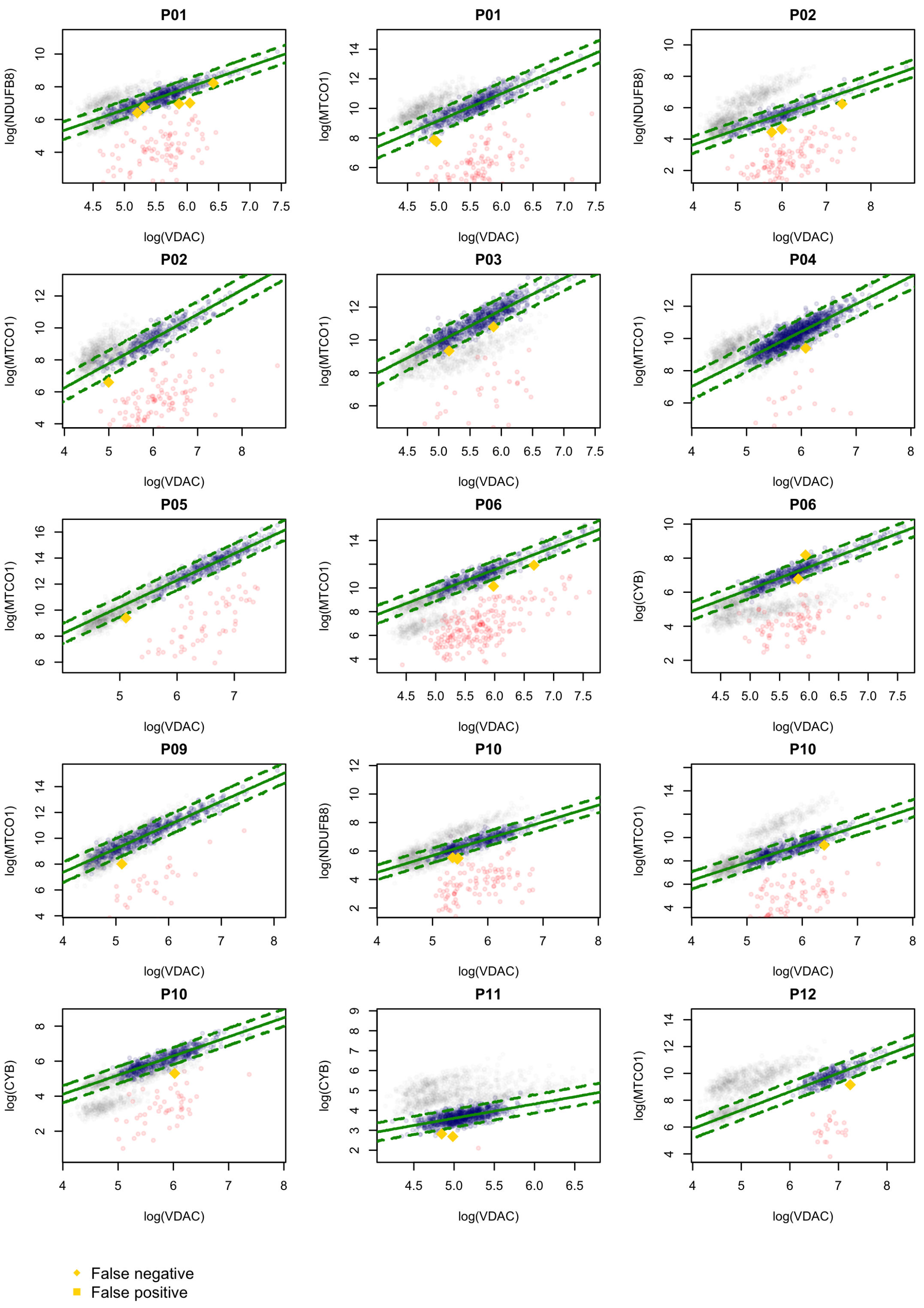

Fig O. Misclassifications of synthetic data D02 are due to data overlap. Synthetic OXPHOS data and the posterior predictive of the Bayesian hierarchical model. Posterior Bayesian classifications are simplified to be whether the posterior expected marginal probability of being not-like-control is above 0.5. Misclassifications are highlighted with a yellow square or diamond, depending on the misclassification type, see legend.
